# Investigating the impact of synonymous gene recoding on a recombinantly expressed monoclonal antibody under different process parameters

**DOI:** 10.1002/btm2.10750

**Published:** 2025-01-27

**Authors:** Nayiri M. Kaissarian, Stephanie L. Sandefur, Arnab Ghosh, Upendra K. Katneni, Wendy Walton, Christopher C. Frye, Anton A. Komar, Chava Kimchi‐Sarfaty

**Affiliations:** ^1^ Hemostasis Branch 1, Division of Hemostasis, Office of Plasma Protein Therapeutics, Office of Therapeutic Products Center for Biologics Evaluation & Research, US Food and Drug Administration Silver Spring Maryland USA; ^2^ Bioproduct Research and Development Eli Lilly and Company Indiana USA; ^3^ Center for Gene Regulation in Health and Disease, Department of Biological, Geological and Environmental Sciences Cleveland State University Cleveland Ohio USA; ^4^ Department of Biochemistry and Center for RNA Science and Therapeutics, School of Medicine Case Western Reserve University Cleveland Ohio USA; ^5^ Genomic Medicine Institute Lerner Research Institute, Cleveland Clinic Cleveland Ohio USA; ^6^ Present address: Integral Molecular Philadelphia PA USA

**Keywords:** CHO cells, codon optimization, monoclonal antibody, process changes

## Abstract

Monoclonal antibodies (mAbs) are commonly used biologic therapeutics with a wide variety of clinical applications. During the development process, manufacturers consider different production parameters to improve protein yield and achieve appropriate quality of the product. Synonymous gene recoding is one of such attributes that is often considered and implemented to enhance protein expression. However, it has to be used with caution, as it may lead to protein misfolding and ER stress, which complicates efforts to manufacture the desired mAb. To investigate how changing mRNA sequence composition under different protein production parameters might affect the quality of recombinantly produced mAbs, we performed a comprehensive and systematic study assessing impact of synonymous gene recoding (commonly referred to as codon optimization) strategies in the context of varied cell culture parameters on product quality, biochemical and functional characteristics. We report the impact of these parameters on mAb glycosylation profiles, charge variant profile, aggregation, fragmentation, and mAb functional response from combinations of different production parameters. These results uncovered a complex interplay of sequence composition and manufacturing parameters and emphasize the importance of assessing changes to key quality attributes when optimizing mAb manufacturing, including the use of synonymous gene recoding.

**Abbreviations**
CAI: codon adaptation index
CDCL: clonally derived cell lines
CHO: Chinese Hamster Ovary
CLF: cell line format
CLG: cell line generation
CO: codon optimized
GCN: relative gene copy number
HC: heavy chain
LC: light chain
mAb: monoclonal antibody
NT: nucleotide
PQA: product quality attributes
PrA: protein A
PS: production scales
RSCU: relative synonymous codon usage
RSCPU: relative synonymous codon pair usage
RI: random integration
TI: targeted integration

**Translational Impact Statement**

During the development of a therapeutic protein, manufacturers may implement process changes that could have unintended consequences on the quality of the therapeutic. This article investigates the impact of some of these process changes on the properties of a monoclonal antibody. The observations from this study reiterate the importance of appropriate comparability studies in the event of significant process changes.

## 1 INTRODUCTION

Monoclonal antibodies (mAbs) are some of the most successful and top (by world‐wide sales) drug products.^1^ Just in 2023, the US Food and Drug Administration (FDA) has approved several mAb‐containing drugs all leveraging Chinese Hamster Ovary (CHO) cells as the production host.^2^ CHO cells are the most often used host, as they can produce mAbs in large amounts and with post‐translational modifications similar to those in human cells.

While many technologies provide high product yield, manufacturers need to maintain safety, efficacy, stability, and desired pharmacokinetics of the recombinant drug product. Thus, changes to manufacturing parameters are routinely introduced throughout pre‐clinical and clinical development and even after approval for marketing. However, maintaining the necessary structural and functional protein properties can be challenging.^3^ One such important product quality attribute (PQA), for example, is the composition of Fc glycans, which can impact many attributes of the therapeutic antibodies.^4^ Therefore, it is important to perform comparability studies to verify the product quality and efficacy.^5^, ^6^

The biopharmaceutical industry often utilizes “codon optimization” (synonymous gene recoding or nucleotide sequence optimization) to improve protein yield. Each optimization algorithm is different, but generally use a set of variable attributes, such as, codon adaptation index (CAI), GC content and/or the complexity/stability of the mRNA secondary structure often reflected by minimum free energy (MFE) values to recode a given sequence.^7^ The underlying assumption during synonymous gene recoding is that synonymous mutations will not affect protein structure and function. However, many recent studies refute this assumption.^8^, ^9^, ^10^, ^11^, ^12^, ^13^, ^14^, ^15^, ^16^, ^17^, ^18^ For example, synonymous recoding of blood coagulation Factor IX has been shown to alter protein conformation and immunogenicity^19^, ^20^ thus raising efficacy and safety concerns.

During the manufacturing process, the desired transgene is usually introduced into (CHO) cells using two main approaches: random integration (RI) or targeted Integration (TI). RI increases the probability of cells expressing multiple copies of the transgene, however during RI, the gene is incorporated into unknown/random location(s) resulting in heterogeneity among cells due to variations in epigenetic properties of the sites of integration. In addition, expression of multiple copies of a gene may lead to endoplasmic reticulum (ER) stress which can further impact the quality of a therapeutic protein.^21^ Thus, TI, which uses site‐specific recombinase‐based CHO cells has recently become a popular choice for recombinant protein production including mAbs.^22^ Nevertheless, one of the remaining issues with CHO cells (that may affect the quality of the expressed protein) is their genomic plasticity and phenotypic diversity. An approach to reduce genetic and phenotypic diversity in transfected cell populations (referred to as a selected bulk culture) is to use clonally derived cell lines (CDCLs) originating from isolated single cells.^23^, ^24^, ^25^ However, CHO CDCLs have been shown to exhibit chromosomal heterogeneity,^26^ resulting in genomic diversity.^27^ Therefore, evaluating genotypic and phenotypic characteristics of CDCLs is important for choosing proper cell lines for production.^23^ There is also an ever‐growing demand for highly productive cellular systems that can be cultured in bioreactors.^28^ As such, it is important to track changes in PQAs when scaling up protein production.^29^

To our knowledge, a comprehensive assessment of the impact of synonymous gene recoding on mAb expression and quality in CHO cells under various production parameters has not been reported. In this study, we have examined the effects of synonymous nucleotide (NT) sequences in the context of different production parameters on the expression and product quality attributes of a recombinantly produced mAb. More specifically, we have compared the effects of synonymous gene recoding using one “native” (NAT) sequence arising from the antibody discovery process and three different synonymous gene recoded transgenes on protein yield, glycosylation, mAb ligand affinity, and many other characteristics. We also compared PQAs among these four different NT sequences, when using two different cell line generation methods (TI and RI), two cell line population formats (Bulk and CDCL), and two different production scales (shake‐flask (SF) and 36L).

Our results show differences in glycosylation profiles, charge variant profiles, their aggregation propensity and fragmentation, and ligand binding characteristics among PrA purified proteins obtained using different manufacturing parameters. Interestingly, various synonymous gene recoding strategies appeared to matter less (in terms of the yield and product quality) than a defined production parameter, revealing previously uncovered attributes underlying distinct mAb manufacturing processes. These data provide important insights to upscale and evaluate the production of functional proteins for medical and biotechnological purposes.

## 2 RESULTS

**FIGURE 1:**
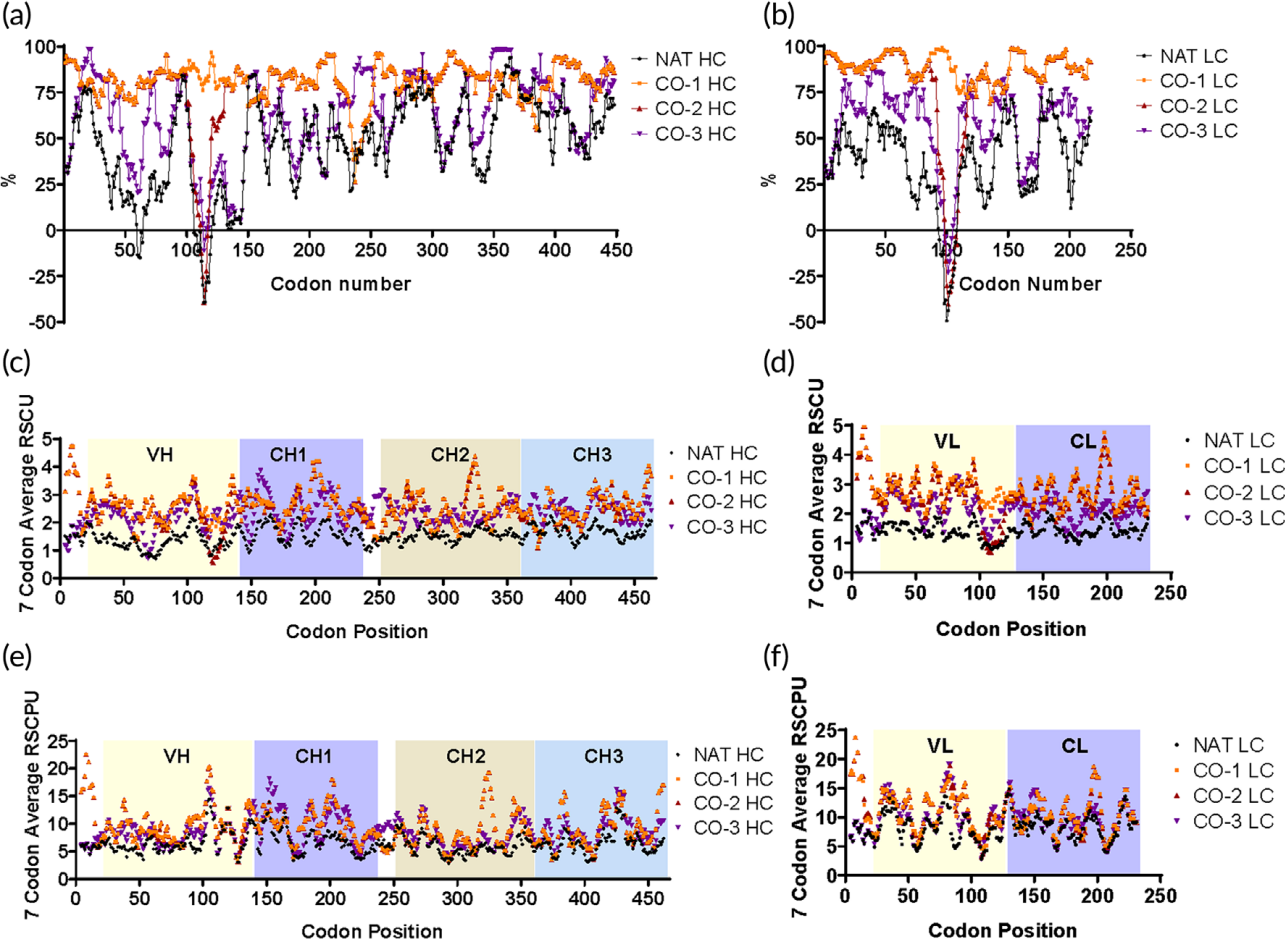
Properties of *mAb1‐IgG4 heavy chain* (*HC*) and *light chain* (*LC*) gene sequences. %MinMax values were plotted by codon position for (a) *HC* and (b) *LC* sequences. Negative and positive values indicate the presence of rarer and more optimized codons, respectively. The seven‐codon average relative synonymous codon usage (RSCU) for (c) *HC* and (d) *LC*, and the seven‐codon average of relative synonymous codon pair usage (RSCPU) were plotted for (e) *HC* and (f) *LC* sequences. IgG4 sequence domains are overlayed on the plots. %MinMax, RSCU, and RSCPU calculations overlap for a majority of *CO‐1* and *CO‐2 HC* and *LC* NT sequences because they only differ by 11 codons per *HC* and *LC* comparison. See also Figures S1 and S2.

We used three “codon optimized” (CO) sequences coding for the same mAb1‐IgG4 mAb to assess changes in IgG4 expression, quality and translation efficiency relative to the NAT sequence. The “*CO‐1*” sequence was designed by GeneArt. The “*CO‐2*” sequence reverted parts of the *CO‐1* sequence back to NAT based on conservation among other species. The “*CO‐3*” sequence was designed to increase GC content while preserving the pace of co‐translational protein folding. Refer to Table 1 for codon adaptation index (CAI) values and GC composition and Table 2 for percent similarities among all *heavy chain* (*HC*) and *light chain* (*LC*) sequences. %MinMax,^30^ seven‐codon average relative synonymous codon usage (RSCU) and relative synonymous codon pair usage (RSCPU) are plotted per codon position in Figure 1. Refer to Figures S1 and S2 for RSCU and RSCPU heatmaps for each codon and codon pair present in the sequences and Figure S3 for NT sequence alignments. *CO‐1* and *CO‐2 HC* and *LC* sequences differ by 11 codons per *HC* and *LC* comparison, which resulted in observable overlap in %MinMax, RSCU, and RSCPU calculations per codon position (Figure 1).

**TABLE 1:** Properties of *mAb1‐IgG4* heavy chain (HC) and light chain (LC) sequences.

Construct	CAI values	GC (%)	MFE (kcal/mol)
NAT HC	0.81	59.01	−535.3
CO‐1 HC	0.94	61.02	−476.4
CO‐2 HC	0.93	60.73	−482.5
CO‐3 HC	0.86	64.45	−547
NAT LC	0.78	52.48	−222.8
CO‐1 LC	0.96	62.13	−232.3
CO‐2 LC	0.94	60.85	−225.2
CO‐3 LC	0.84	60.85	−241.3

*Note*: Codon adaptation index (CAI) values, and GC content of the native (NAT) and three different codon optimized (CO) heavy chain (HC) and light chain (LC) sequences. Minimum free energy (MFE) was calculated for each HC and LC synonymous sequence.

**TABLE 2:** Nucleotide and codon sequence similarities.

		NAT	Nucleotides		
			CO‐1	CO‐2	CO‐3
Heavy chain					
	NAT		82.26	83.12	88.77
Codons	CO‐1	54.08		99.14	86.91
	CO‐2	56.44	97.64		87.05
	CO‐3	75.75	64.38	64.81	
Light chain					
	NAT		78.72	80.28	84.96
Codons	CO‐1	47.23		98.44	83.69
	CO‐2	51.91	95.32		84.11
	CO‐3	67.66	62.55	64.26	

*Note*: Percent alignment of the nucleotides (blue) and percent of codons remaining unchanged (green) among the four HC and LC synonymous sequences. See also Figure S3.

### 2.1 Cell‐free experiments reveal effects of synonymous gene recoding on mAb translatability

**FIGURE 2:**
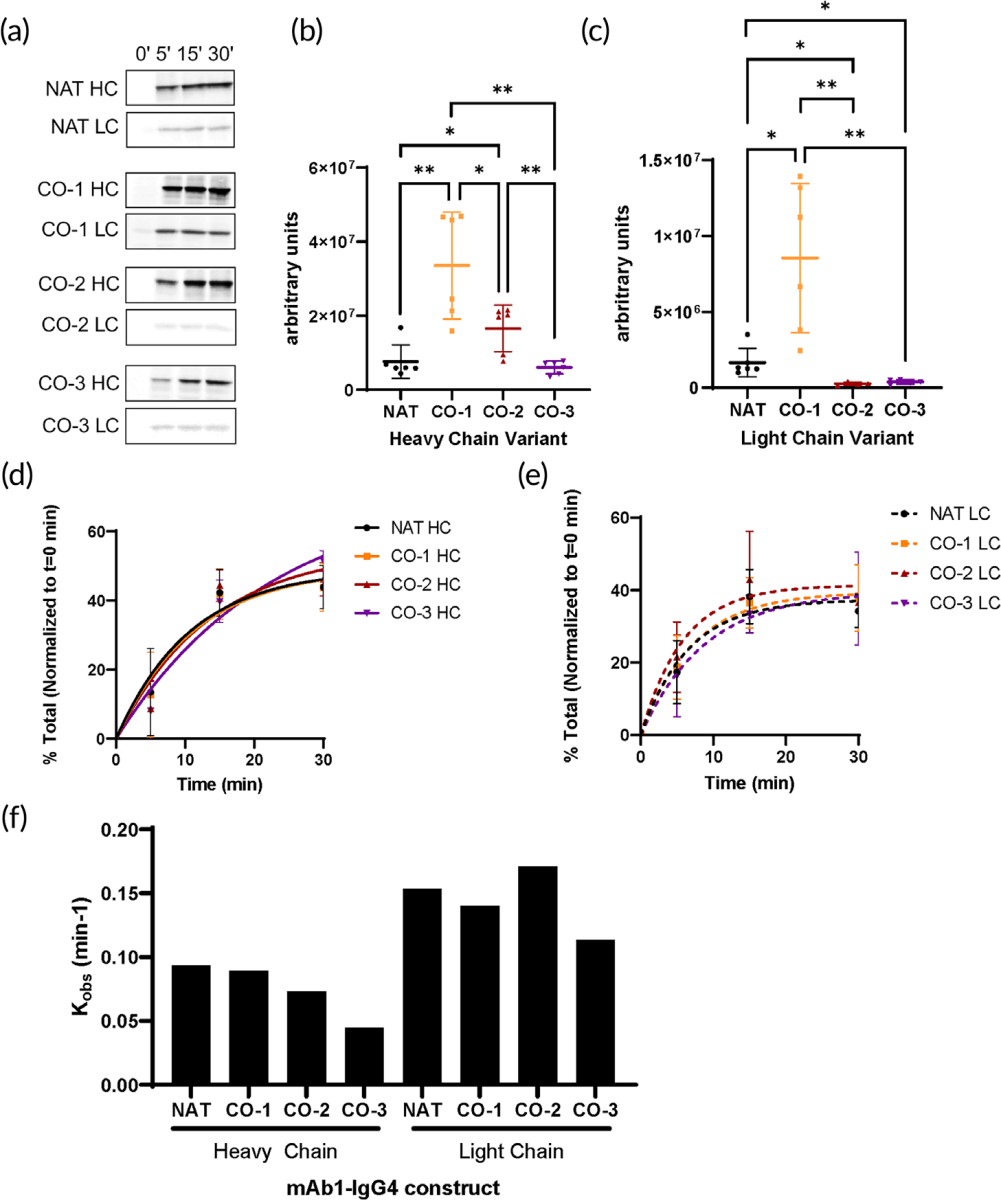
Translation Kinetics of three different codon optimized (CO) *IgG4* heavy chain (HC) and light chain (LC) compared to the native (NAT) sequence. (a) Representative radiograph of the translation rate (^35^S‐Met incorporation). (b) and (c) The arbitrary values obtained from phosphor imaging scans for products formed after 30 min were used to plot the scatter plot showing expression differences of (b) heavy chain (HC) and (c). light chain (LC). Bars represent mean ± SD. Each symbol represents one replicate. Two‐tailed Welch's *t* test. The arbitrary values obtained from phosphor imaging scans in Figure 1a were used to plot expression differences at 0, 10, 20, and 30 min of (d) HC and (e) LC. Bars represent mean ± SD. Each symbol represents one replicate. F. One phase exponential association curve fitting was used to calculate kinetics parameters of the in vitro translation reaction. See Appendix S2 for more details on statistics and curve fitting.

We first compared the four NT sequences above using an in vitro cell‐free system aiming to assess the impact of synonymous gene recoding on protein translation without the underlying effects of the cellular environment. We generally and expectedly found that sequences with higher CAI (*CO‐1* and *CO‐2*, Table 1) resulted in higher overall yield of the in vitro translation products compared to NAT, while the *CO‐3* sequence with only marginally increased CAI (over NAT) generally showed similar (to NAT) expression levels. The *CO‐1* sequence (HCs and LCs) with the highest overall CAI had a greater expression after 30 min compared to the other sequences (Figure 2a–e). These expression differences however did not correlate with the predicted MFE differences among the sequences (Table 1). Higher MFE generally is expected to slow ribosome movement.^31^ Interestingly, different sequences revealed relatively similar translational dynamics (as reflected in *K*_obs_ values) except for the *CO‐3* HC construct that had lowest *K*_obs_ indicating a decreased rate of product formation over time and a delay in reaching the translational plateau (Figure 2f).

### 2.2 Integration method and cell line format influence mAb yield more than a defined synonymous gene recoding approach

**FIGURE 3:**
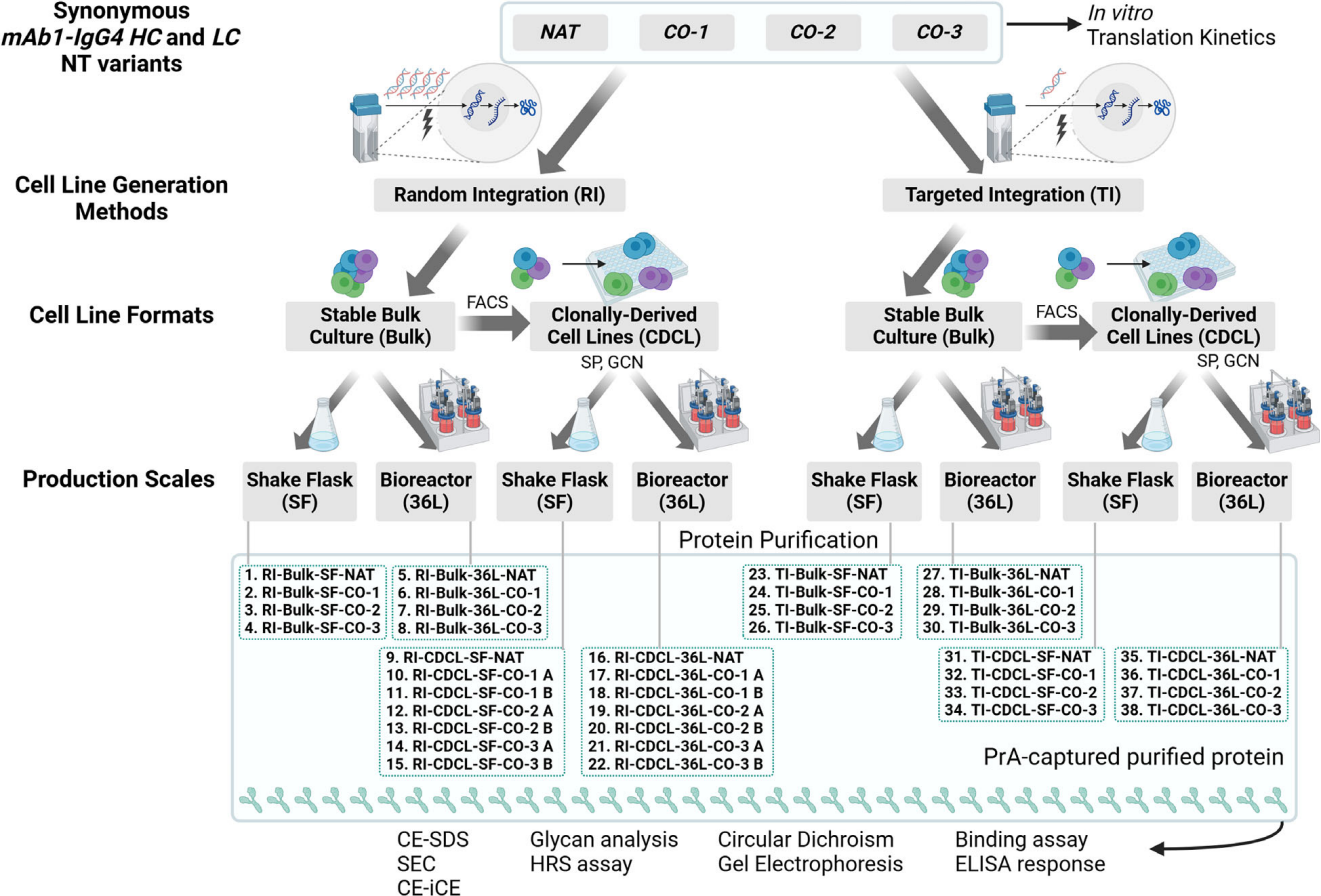
Schematic of methods used to manufacture material used for analysis. The Native (NAT) and three codon optimized (CO‐1, CO‐2, CO‐3) mAb1‐IgG4 heavy chain (HC) and light chain (LC) nucleotide (NT) sequences were transfected by electroporation into CHO cells by random (RI) or targeted integration (TI). Stable bulk culture (bulk) cells were sorted into single‐cell colonies by FACS. Clonally‐derived cell lines (CDCLs) were chosen based on specific productivity (SP) and gene copy number (GCN) for further evaluation. Bulk and CDCL cells were grown in two production scales, shake flask (SF) and 36L. Protein A (PrA)‐captured purified protein from all groups were analyzed by the methods listed at the bottom of the figure. This figure was created with BioRender.com.

We further used the CHO cells to assess the impact of synonymous gene recoding and production methods/process parameters on protein yield and protein attributes. The process parameters included two cell line generation methods (TI and RI), two cell line formats (Bulk and CDCLs), and two different production scales (SF and 36L). In total, there were 32 different combinations of these four variables: NT sequence, cell line generation (CLG) method, cell line format (CLF), and production scale (PS). The NT sequence optimized RI‐CDCL groups had two cell lines each that were suitable (based on productivity) for use for further protein production. Therefore, we have analyzed 38 recombinantly produced protein samples (Figure 3). For each analytical method, results were combined for samples that shared one, two, or three of the variables. Many measurements had slight, yet consistent differences between groups (see Tables S1–S9 for all attributes). Notable differences are described below.

**FIGURE 4:**
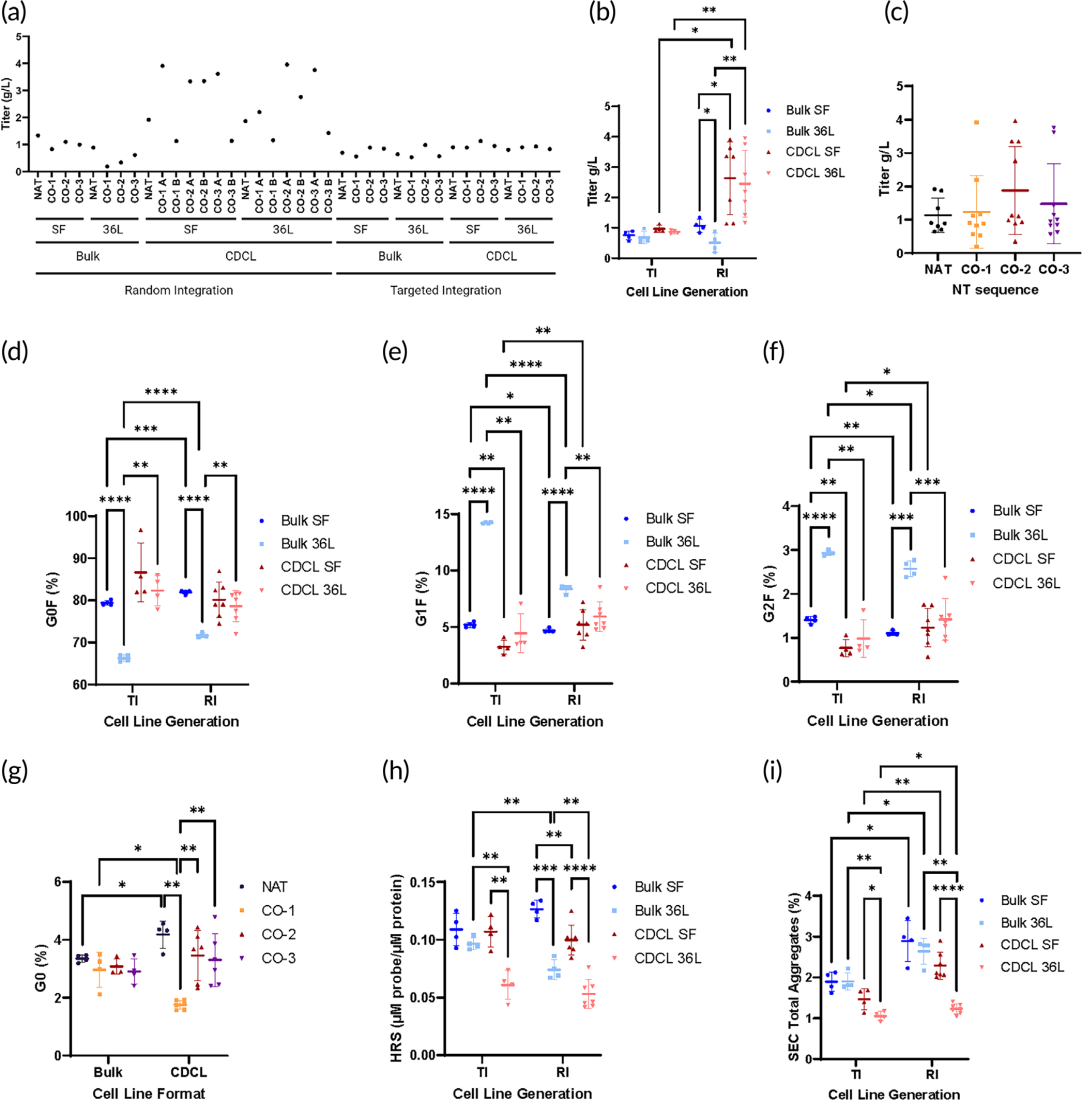
Synonymous gene recoding and process parameters affect product quality attributes (PQAs). Higher titer is observed from codon optimized RI‐CDCLs reported by (a) each IgG4 protein sample, (b) grouping the different CLG‐CLF‐PS variables, and (c) grouping the different NT sequences. Observations made from glycan analysis include PS and CLF affected (d) G0F content, (e) G1F content, and (f) G2F content regardless of CLG method. (g) Codon optimization affected G0 content among protein purified from CDCLs but not stable bulk culture cells. (h) Differences in carbonyl type oxidation is observed from the hydroxylamine species (HRS) assay. (i) Changes in total aggregation is observed by SEC. In both integration methods, less aggregation is observed in CDCL‐36 L than other groups. Except for CDCL‐36L, higher aggregates are observed in RI than TI. Bars represent mean ± SD. Each symbol represents one sample. See Appendix S2 for details from Welch's *t* tests for Figure 3b,d–i. See also Tables S1–S9. SEC, size exclusion chromatography.

While the *CO‐1* sequence showed the highest average relative yield in a cell‐free system (Figure 2b,c), the same trend was not observed in a cellular system when comparing mAb titers after purification via protein A (PrA) capture from CHO cells using various production schemes. We found that NT sequence per se was not the major factor affecting average titer (Figure 4c). Out of the top 16 producers of the 38 samples, 15 were RI‐CDCLs and were comprised of all four NT sequences (Figure 4a–c). The higher productivity in these RI‐CDCLs could be explained by the higher relative gene copy number (GCN) compared to the other CDCLs (Appendix S1).

### 2.3 Synonymous gene recoding and process parameters affect mAb glycosylation

**FIGURE 5:**
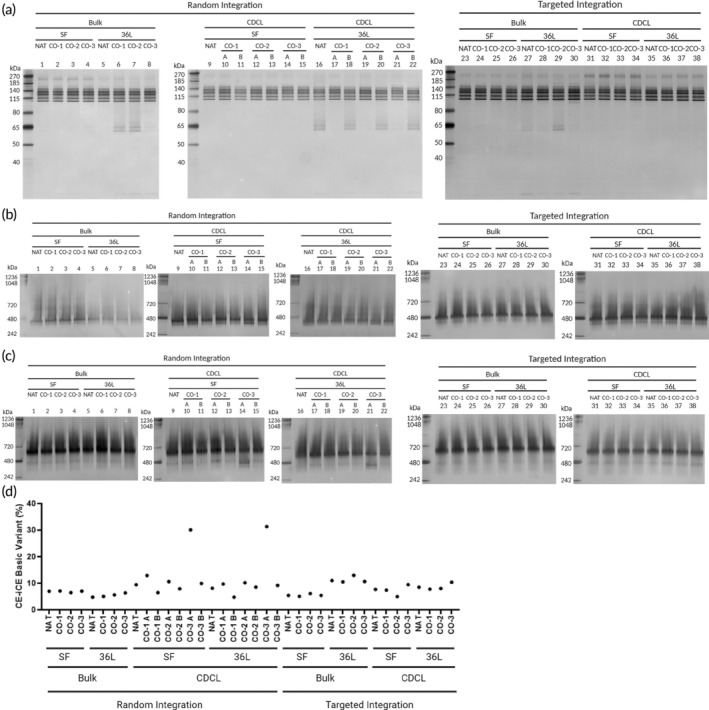
Composition of mAb1‐IgG4 purified protein. (a) Random integration (RI) and targeted integration (TI) samples were analyzed by nonreducing SDS‐PAGE followed by silver staining. A strong band is observed between 185 and 270 kDa in all samples in the RI‐bulk‐SF (samples 1–4) and TI‐CDCL‐SF (samples 31–34) groups. Stronger fragments are observed <65 kDa in: two CO samples from the RI‐Bulk‐36L group, select samples from the RI‐CDCL‐36L group, and the NAT and CO‐2 samples from the TI‐Bulk‐36L group. It is observed that protein composition is not consistent among protein manufactured from different synonymous nucleotide sequences within these groups. RI and TI samples were separated by NativePAGE in (b) dark blue cathode buffer (0.02% G‐250) for ~1/3 of the gel following by light blue cathode buffer (0.002% G‐250) for the remainder of the gel or in (c) light blue cathode buffer (0.002% G‐250) only, followed by silver staining. Samples 14 and 21 are from the shake flask and 36L groups, respectively, but are from the same cell line (RI‐CDCL‐CO‐3 A) and exhibit stronger bands than other samples at ~480 kDa when separated by light blue cathode buffer (panel c). The same is not observed for the other cell line of the same variables (RI‐CDCL‐CO‐3 B, panel c samples 15 and 22). (d) RI‐CDCL CO‐3 A in shake flask and 36L production scales is observed to have a higher percentage of basic variants (30.11% and 31.35%, respectively) than RI‐CDCL CO‐3 B, which falls within the range of the other samples (4.74%–12.97%). See also Figure S5.

Glycosylation may affect biological activity, immunogenicity, and cytotoxicity, and is an important PQA to monitor among manufactured mAbs.^32^ We observed substantial differences in N‐linked glycosylation among CLG, CLF, and PS groups. G0F comprises the majority of the glycans found on these mAbs (Figure 4d). Bulk‐36L in both RI and TI groups tends toward more complex glycosylation—G1F and G2F (Figure 4e,f), rather than G0F (Figure 4d) compared to other CLF and PS (RI and TI) groups. Effects of synonymous gene recoding on prevalence of the N‐linked glycosyl group, G0, were also observed (Figure 4g). Interestingly, in the CDCL group, the *CO‐1* sequence revealed the lowest average G0 glycan content in comparison with NAT and other sequences (Figure 4g). At the same time, no significant differences were observed among the four NT sequences in the Bulk group (Figure 4g). In addition, lower G0 glycan content was observed from the *CO‐1* sequence in the CDCL format than the Bulk format. In contrast, higher G0 glycan content was observed from the *NAT* sequence in the CDCL format than in the Bulk format (Figure 4g). In addition to the NT sequence differences within the CDCL group observed in G0 glycostructure comparisons, the CDCL group had a wider range of G0 (1.57%–4.79%) than the Bulk group (2.12%–3.49%) (Figure 4g). However, all these observed differences may fall within the method variability (~3%–5%). In addition, when samples were separated by nonreducing SDS‐PAGE, multiple ~115–140 kDa bands representing different glycosylated forms were detected (Figure 5a). RI‐CDCL samples exhibited variations between proteins from RI‐CO samples. The relatively faint top band of the monomer was more abundant in CDCL‐CO‐3 A than B samples in both production scales (Figure 5a, samples 14 and 21 compared with 15 and 22). This difference was not noted for other CO sequences, appearing to be unique for CO‐3.

### 2.4 Process parameters affect carbonyl type oxidation

The hydroxylamine reactive species (HRS) assay measures carbonyl type oxidation in proteins.^33^ Although NT sequence variations did not impact HRS abundance (Table S7), there were observable differences among different CLG, CLF, and PS groups (Figure 4h). All TI‐CDCL‐36L, RI‐Bulk‐36L, and RI‐CDCL‐36L proteins were below the industry benchmark (0.1 μM probe/μM protein). All RI‐bulk‐SF samples were found to be above the benchmark. CDCL‐36L samples had the lowest mean HRS in both TI and RI groups. Within both TI the RI groups, Bulk‐36L protein measured higher than CDCL‐36L. Bulk‐SF averaged higher than CDCL‐SF in the RI group, but not in the TI group. Within the TI group, Bulk‐36L protein averaged similarly to the Bulk‐SF and CDCL‐SF proteins.

### 2.5 Gene integration methods influence mAb aggregation and oligomerization propensities

For each CLF‐PS group, higher total aggregates were observed in RI than TI (Figure 4i). Within the TI and RI groups, similar total aggregates were observed between Bulk‐SF and Bulk‐36L and lower aggregates were observed in CDCL‐36L groups compared to CDCL‐SF and Bulk‐36L groups (Figure 4i). More oligomers (>185 kDa) were observed in samples from the RI‐Bulk‐SF and TI‐CDCL‐SF (Figure 5a) groups than the other CLG‐CLF‐PS groups on nonreducing SDS‐PAGE gels. Additional differences in oligomerization were observed ~720 kDa when gels were separated first in 0.02% G‐250 followed by 0.002% G‐250 (Figure 5b) rather than 0.002% G‐250 alone (Figure 5c). Within the RI group, the strongest oligomer bands were observed among Bulk‐SF samples (Figure 5c). Within the TI group, stronger oligomer bands were observed in CDCL samples than Bulk samples (Figure 5c). This observation is in line with observations between these two groups on nonreducing SDS‐PAGE (Figure 5a).

### 2.6 Effects of synonymous gene recoding on mAb fragmentation and charge

Differences in mAbs fragmentation (compare bands <80 kDa) were observed in nonreducing SDS‐PAGE gels. Overall, more fragments were observed in samples from the RI‐bulk‐36L, RI‐CDCL‐36L, and TI‐Bulk‐36L groups than from the TI‐CDCL‐36L and all SF groups (Figure 5a). Within the RI‐CDCL group, 36L samples revealed more variation among pairs from the same NT sequences than SF samples (Figure 5a). Within the RI group, all CO CDCL samples had two CDCL samples each. From the six possible comparisons between samples from the same CO sequence and cell culture method, three had consistency between CDCL pairs (Figure 5a, sample numbers CO‐1 10/11, CO‐2 12/13, CO‐3 14/15, all from the SF group, no fragments observed). Fragments are observed for one CDCL from each pair from each 36L‐CO NT sequence (Figure 5a, sample numbers CO‐1 17/18, CO‐2 19/20, and CO‐3 21/22). In addition, the single sample from CDCL‐36L‐NAT (16) also has fragments (Figure 5a).

In accordance with observations from nonreducing SDS‐PAGE, variation among pairs from the same NT sequences within the RI‐CDCL group was also observed on NativePAGE gels separated by 0.002% G‐250 only. Variation between RI‐CDCL pairs from the same NT sequences was also observed for RI‐CDCL CO‐1 and CO‐2 samples on NativePAGE gels. CDCL‐CO‐1 A has a stronger band below the main monomer band (~480 kDa, Figure 5c SF and 36L, samples #10 and #17) than the other CO‐1 B (Figure 5c SF and 36L, samples #11 and #18). Similarly, CDCL‐CO‐2 A (Figure 5c sample #12) had a stronger band below monomer than CO‐2 B (Figure 5c sample #13). Notably, CDCL‐CO‐3 A (Figure 5c, SF and 36L, sample #14 and 21) had two strong observable bands ~480 kDa that are not as prevalent in the other sample from the same RI‐CDCL NT sequence, CO‐3 B (Figure 5c, SF and 36L samples #15 and 22) or the other proteins on the gels (Figure 5c).

In addition, a higher relative abundance of basic variants was observed via CE‐iCE from RI‐CDCL‐CO‐3 A (SF and 36L) protein compared with all other samples, in both production scales (Figure 5d). These results suggest that the unique combination of using the CO‐3 sequence along with CDCL format, regardless of production scale, affects charge variants present in the purified protein product.

### 2.7 Process parameters affect relative response in ELISA experiments

**FIGURE 6:**
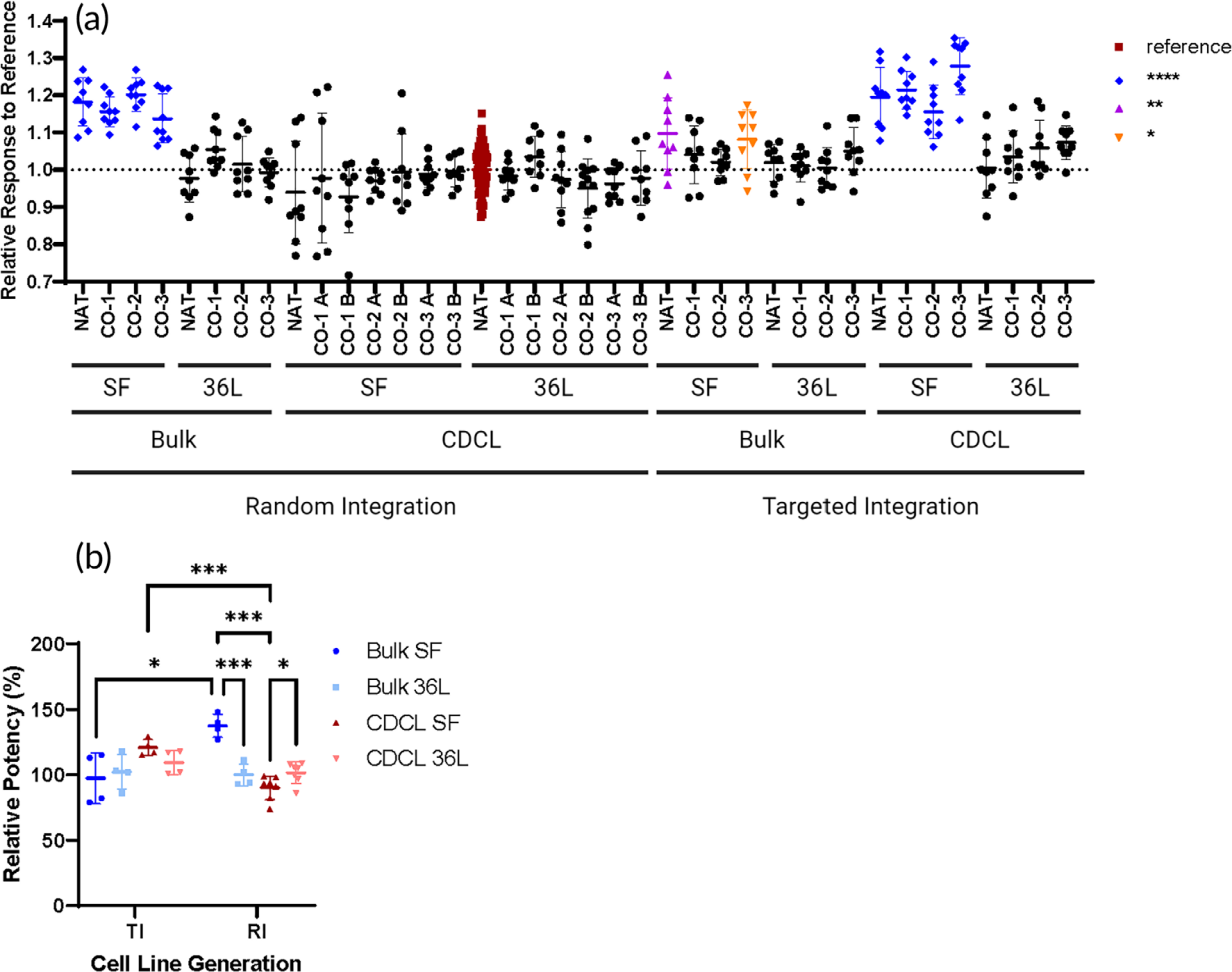
Process parameters affect efficacy and potency of mAb1‐IgG4 in ELISA. (a) The relative response at the highest dose of mAb1‐IgG4 was calculated for each sample relative to the response of the reference standard, RI‐NAT‐CDCL‐36 L. The RI‐Bulk‐SF and TI‐CDCL‐SF samples have significantly higher relative response than the reference (*p* < 0.0001) as observed on the dose–response curves. These samples also have a strong presence of an oligomer on nonreducing SDS‐PAGE gels (Figure 4a,b). The means of TI‐Bulk‐SF‐NAT (*p* = 0.0017) and TI‐Bulk‐SF‐CO‐3 (*p* = 0.021) were also found to be statistically higher than the reference (one‐way ANOVA with Dunnett's multiple comparisons test). Bars represent mean ± SD. Each symbol represents one replicate. (b) The RI‐Bulk‐SF group had higher average relative potency than RI‐bulk‐36L, RI‐CDCL‐SF, and TI‐Bulk‐SF groups (Welch's *t* tests). The difference between the means of RI‐CDCL SF and 36L groups while significant, are within 80%–120% method variability. Bars represent mean ± SD. Each symbol represents one sample. See Appendix S2 for details on all statistical tests performed. See also Figures S4 and S6.

No substantial changes were observed in substrate binding constants among the different NT sequences by Surface plasmon resonance (SPR) (Figure S4a–c). However, there were notable differences observed in dose–response curves from ELISA data. Samples from the TI‐CDCL‐SF and RI‐Bulk‐SF groups had greater upper asymptotes compared to the reference sample (Figure S6). These differences are apparent when calculating relative response to the reference sample at the highest dose (Figure 6a). These same two groups of samples are the same groups that exhibit a strong band ~270 kDa on nonreducing SDS‐PAGE gels (Figure 5a). Relative potency was calculated from the ELISA data by using a constrained fit (described in methods). RI‐Bulk‐SF had higher relative potency than TI‐Bulk‐SF, RI‐Bulk 36L, and RI‐CDCL‐SF (Figure 6b). TI‐CDCL‐SF had a higher relative potency than RI‐CDCL‐SF (Figure 6b). RI‐CDCL36L had a slightly higher mean relative potency than RI‐CDCL‐SF, but both means are within 80%–120% method variability (Figure 6b).

## 3 DISCUSSION

mAbs are manufactured to treat a wide variety of diseases from cancer to SARS‐CoV‐2^34^ and optimization and production processes must ensure product safety, efficacy and stability. While different manufacturing processes can be used, CDCLs are prevalent due to the assumption of minimal heterogeneity for the purposes of consistent product quality,^35^ high yield and the compliance with the current regulatory requirements. However, heterogeneity may still exist within CDCLs, and differences between CDCLs originating from the same bulk cell population can be drastic.^27^ The observed differences between the CO‐1, CO‐2, and CO‐3 CDCLs from the RI‐CDCL group (Figure 5a) clearly suggest that CDCLs must be screened rigorously as they can impact the quality of the biotherapeutic.

One of the drawbacks of CDCLs it the timing required for cell line generation and subsequent screening based on desired characteristics.^35^ To accelerate production timelines, stable bulk cultures can be used and no substantial differences in product quality were previously observed between proteins manufactured using stable bulk culture cells and CDCLs.^34^, ^36^, ^37^ However, here we observed notable differences between proteins manufactured via stable bulk culture and CDCLs (Figures 4, 5, 6).

Significant changes in the relative abundance of N‐linked glycosyl groups, G0F, G1F, and G2F (Figure 4d–f) among the different CLG, CLF, and PS groups were found. Notably, the Bulk‐36L group, regardless of integration method, exhibited a propensity toward more complex glycosylation, suggesting that a defined production method should be considered given its potential impact on protein glycosylation. We also observed small variations in the nonfucosylated G0 glycostructure among the CDCLs from different CO sequences, but not among Bulk (Figure 4g). A recent study also reported variations in glycosylation between non‐CO and CO IgG3.^38^ Together these studies expose the potential effects of synonymous mutations on antibody quality. However, as we found, such effects may depend on a defined production method rather than sequence optimization approach.

We further observed small, yet measurable differences in aggregation propensity among different CLG, CLF, and PS groups. Regardless of integration method, CDCL‐36L samples revealed lower aggregation compared to other groups. RI has higher total aggregates than TI, except for RI‐CDCL‐36L which has similar aggregation to TI‐CDCL‐36L (Figure 4i). As aggregation may affect immunogenicity of a therapeutic protein,^39^ this finding may further inform choice of production parameters.

Antibodies often have basic isoelectric points and are stable in acidic formulations.^40^ However, changes in the charge may affect the half‐life and efficacy of biotherapeutics^41^ and a higher pI may increase clearance of the therapeutic antibody.^42^ Interestingly, RI‐CO‐3 CDCL A samples had the highest percentage of basic variants as analyzed by CE‐iCE (Figure 5d). The same samples also revealed fragments on NativePAGE (Figure 5c) in two different production scales. The observation that both samples from different production scales (SF and 36L) from the same CDCL revealed heterogeneity underlines another important aspect of the relationship between synonymous gene recoding, production methods, and product quality attributes.

We did not observe substantial changes among all samples in far‐UV circular dichroism (CD) studies (Figure S5) or among TI samples in near‐UV CD (data not shown) and differential scanning calorimetry (data not shown). We also did not observe changes in substrate affinity among all samples employing SPR measurements (Figure S4). However, ELISA experiments did reveal apparent differences among protein produced using different process parameters (Figure 6a,b). Eight samples had higher upper asymptotes observed on dose response curves (Figure S6). The same eight samples from the TI‐CDCL‐SF and RI‐Bulk‐SF groups also exhibited oligomers on nonreducing gels (Figure 5a). Oligomerization could affect substrate binding during ELISA. However, oligomerization observed during gel‐electrophoresis may not necessarily correlate with oligomerization in physiological conditions.^43^

A challenge for manufacturing the desired mAb is using an expression system that correctly folds and modifies the expressed protein. Enhanced protein expression due to synonymous gene recoding may lead to protein misfolding and ER stress.^21^, ^28^ In our study, despite observations of differences in product quality, we did not observe substantial differences in protein folding/structure as evaluated by CD spectroscopy (Figure S5), which admittedly is unable to determine subtle changes.

## 4 CONCLUSION

In sum, our study suggests a complex interplay of synonymous gene recoding strategies and manufacturing parameters influencing mAb production attributes, revealing previously uncovered characteristics that can impact mAb quality/traits. These results reaffirm the need to demonstrate comparability of product manufactured after introduction of process changes, including the use of synonymous gene recoding. Ultimately any differences seen would need to be assessed in terms of risk to clinical performance of a given product.^44^ However, our study contributes to better understanding of the relationship between protein manufacturing parameters and synonymous gene recoding and gives insights to upscale and evaluate the production of functional proteins for medical and biotechnological purposes. The multiparameter screening approach presented in this study allows for rapid identification of factors, which influence key attributes during the manufacturing of mAbs.

## 5 MATERIALS AND METHODS

### 5.1 Nucleotide sequences

The mAb1 molecule is a humanized IgG4‐variant mAb with neutralizing activity against ligand‐mediated Hedgehog pathway activation. The original sequence derived through the antibody discovery process is designated “native” or “NAT.” Three different synonymous gene recoding “codon optimization” (CO) approaches, “CO‐1,” “CO‐2,” and “CO‐3,” were utilized to design *IgG4 heavy chain* (*HC*) and *light chain* (*LC*) DNA sequences and compared to “*NAT*.” *CO‐1 HC* and *LC* were optimized by GeneArt (Regensburg, Germany now a part of Invitrogen, Carlsbad, CA) using their proprietary GeneOptimizer™ technology.^45^ The optimization process included the following: the codon usage was adapted to the codon bias of CHO genes. In addition, regions of very high (>80%) or very low (<30%) GC content were avoided where possible. The following *cis*‐acting sequence motifs were avoided: internal TATA‐boxes, chi‐sites, and ribosomal entry sites; AT‐rich or GC‐rich sequence stretches; ARE, INS, and CRS sequence elements; repeat sequences and RNA secondary structures; (cryptic) splice donor and acceptor sites and branch points. The *CO‐2 HC* and *LC* sequences were designed by reverting 11/455 *CO‐1 HC* codons and 11/224 *CO‐1 LC* codons to the *NAT HC* and *LC* sequences, respectively. These codons were chosen based on their high conservation among various species. *CO‐3 HC* and *LC* were designed using a proprietary algorithm generally aimed at targeted increase of GC content that would nevertheless preserve the natural translation rhythm of the mRNA, ensuring correct co‐translational protein folding. All four HC and four LC NT sequences encode for the same HC and LC primary amino acid sequences, respectively.

### 5.2 In vitro translation

For in vitro expression, plasmid DNAs were linearized using *Eco*RI‐HF (NEB) and cleaned up using DNA Clean & Concentrator kit (Zymo Research). mMESSAGE mMACHINE T7 Ultra kit (Invitrogen) was used to synthesize mRNAs in the presence of ARCA‐cap (Anti‐Reverse Cap Analog). Reaction was incubated at 37°C for 2 h. After incubation, Turbo DNase was added to each reaction and incubation was continued at 37°C for additional 15 min. mRNAs were further purified using MEGAclear Kit (Invitrogen). The integrity of RNA samples was verified using 1% Agarose MOPS/Formaldehyde gel containing ethidium bromide. In vitro translation reactions were carried out using the Rabbit Reticulocyte Lysate (RRL) System (Promega). RNA samples (0.5 μg each) were diluted in total 10 μL nuclease free water and added to the reaction mixture containing 35 μL RRL, 1 μL amino acid mix w/o Met, 3 μL [^35^S]‐Met and 1 μL RNasin (40 U/μL). Reactions were incubated at 30°C. Five microliters of reaction mix were aliquoted at each time interval (0, 15, 30, 60 and 90 min) and added to 20 μL loading dye, boiled at 100°C for 2–3 min and resolved on 12.5% SDS‐Polyacrylamide gel. Gels were dried, exposed to phosphor imaging screen, and analyzed using Typhoon 5 imager. All experiments had two biological replicas each consisting of three technical repeats (six samples overall). To understand the rate of protein synthesis corresponding to the unique sequences, we measured the final translation product formed over time. The data was plotted as % Total vs. time and fitted to a single exponential rate equation *y* = A*[1 − exp(−*K*_obs_*t)] where *A* = amplitude, *t* = time, *K*_obs_ = observed rate constant, and *y* = % total product formed.^46^ This single exponential rate equation describes the relationship between the translation activity (measured here as product formed and, expressed as % Total) over time.

### 5.3 Raw materials and cell culture media

All molecular biology techniques required for this study were carried out utilizing standard methods, including DNA cloning, and protein expression evaluations and analyses. Crude quantitative and qualitative protein expression analyses were carried using the Octet (Pall ForteBio Corp., Menlo Park, CA), according to the manufacturer's protocol. Cells from a previously described^47^ glutamine synthetase knockout (GS_KO)^47^ CHOK1SV cell line (Lonza Biologics, Slough, UK) were cultured using Parental Maintenance Medium in shake‐flasks at 36.5°C, 6% CO_2_, and shaken at 125 rpm, unless otherwise noted. Passages of the parental cell culture were performed on an alternating 3‐day/4‐day schedule. Each transfection was performed in duplicate.

### 5.4 Cell line generation—targeted Integration

TI was performed using a GS_KO‐CHOK1SV parental cell line that contained a Cre landing pad. Transfection was performed using the Nucleofector® (Basel, Switzerland) and the Lonza Amaxa cGMP Nucleofector Kit V. Each transfection cuvette containing 1.5E+6 viable cells (vc) was pelleted and resuspended in the provided Nucleofector Solution provided in the kit. 1.5 μg expression plasmid (*NAT*, *CO‐1*, *CO‐2*, or *CO‐3* incorporated into a proprietary vector containing a G418 selection marker) was added to the cuvette. Post‐electroporation, cells go through selection by removal of glutamine from the medium. If the Cre‐mediated recombination event occurs, the cells will re‐constitute the GS gene, and will survive in medium lacking glutamine (selection medium). Following transfection, the cells were transferred to shake‐flasks containing 20 mL of selection medium. The flasks were placed in a 37°C incubator, shaking at 60 rpm. Cells were counted and pellet/passed at 0.2E+6 vc/mL on a 3‐day/4‐day passage schedule until viability reached >90%. On day 18, cells were pellet/passed at 0.2E+6 vc/mL in selection medium and 800 μg/mL G418 on a 3‐day/4‐day schedule until viability reached >95%. The G418 selection is to ensure that the cells that survived the GS selection did not “kick out” the antibody *LC* and/or *HC* genes. Cells that survive both GS and G418 selection have a higher probability of containing the LC and HC genes. Once cells were recovered from selection, the selected bulk culture was single‐cell sorted and evaluated in a shake‐flask terminal study.

### 5.5 Cell line generation—random integration

RI was performed using GS_KO‐CHOK1SV parental cell line. Three days prior to electroporation, the parental maintenance culture was scaled up by seeding an appropriate number of flasks each containing 125 mL of Parental Maintenance Medium at a vc density of 2.0 × 10^5^ cells/mL and a viability >95%.

Electroporations were performed using a set‐up that included a Gene Pulser XCell, PC Module, and a CE Module (BioRad Laboratories, Hercules, CA). For each electroporation, the linearized plasmid DNA (5 μg) was aliquoted into an electroporation cuvette containing the CHO parental cells (20 × 10^6^ vc). Electroporation conditions were 300 V and 1000 μF capacitance (resistance ∞, gap 4 mm). For the mock transfections, sterile water equivalent to the volume that was used for the transfections containing DNA was combined with the GS_KO‐CHOK1SV cells in an electroporation cuvette. There were two electroporations for each plasmid, as well as two electroporations for the mock.

Post‐electroporation, the contents from the duplicate cuvettes of each expression plasmid, or control, was added to individual E125 shake‐flasks containing 25 mL of the Selection Medium. All flasks were incubated at 36.5°C, 6% CO_2_ and held static for 2 h, after which they were shaken at 125 rpm.

The day following electroporation, each culture's density and viability were determined via Vi‐Cell. The cells from the replicates of each electroporated culture were pelleted via centrifugation and the recovery media decanted. The cells from each culture were seeded at a volume such that the initial seed density was between 2.0 and 4.0 × 10^5^ vc/mL in Selection Medium. Throughout bulk culture selection, on a 3‐day/4‐day passage schedule, the cultures were counted via Vi‐Cell. Each culture was then pelleted, the supernate decanted, and the cell pellet suspended in the same initial volume of Selection Medium, regardless of the resulting seed density. The incubation conditions for bulk selection were 36.5°C, 6% CO_2_, and shaken at 125 rpm. When the selected bulk culture viability reached >95%, they were single‐cell sorted and evaluated in a shake‐flask terminal study.

### 5.6 Cell line format—bulk culture

Selected TI and RI bulk cultures were evaluated in a 14‐day fed‐batch shake‐flask study. On the day of inoculation, the selected bulk culture was counted via Vi‐Cell to determine the cell density and viability. The appropriate amount of culture needed for the terminal study (0.75 × 10^6^ vc/mL in 40 mL) was transferred to a centrifuge tube and pelleted. An aliquot of the cell‐free supernatant (2.5 mL) was removed via pipette and held in reserve. The remaining cell‐free supernatant was decanted from the centrifuge tube, and the cell pellet was suspended in the conditioned medium previously removed and held in reserve. Following suspension of the cell pellet, the cells (5 mL) were added to an E125 flask containing 37.5 mL of the Expression Medium. Cultures were incubated at 35°C and 6% CO_2_ and were shaken at 165 rpm. Due to high cell densities (10–17 × 10^6^ vc/mL) on day seven of the terminal study, the temperature was shifted to 33°C. All flasks received nutrient feeds on days 4, 7, and 10. In addition to the nutrient feed, the flasks also received a tyrosine feed and a glucose feed, if necessary, to maintain a level > 20 mM. On days 4, 7, 10, 12, and 14 each culture was counted via Vi‐Cell and samples were taken for analysis via the Nova bioanalyzer. On days 10, 12, and 14, samples were collected, pelleted, and the supernates stored at −20°C for initial product concentration assessment via PrA chromatography.

### 5.7 Cell line format—clonally derived cell lines

CDCLs were generated using fluorescence‐activated cell sorting (FACS)‐based single‐cell sorting of TI and RI Bulk culture cells for each NT sequence. Bulk culture samples were sorted once they had recovered to above 80% viability (Vi‐Cell). Prior to sorting, 96‐well plates (10 plates/selected bulk culture) were filled using the Wellmate. The selected bulk cultures were single cell sorted via FACS (MoFlo, DakoCytomation). Single, viable cells were identified by measuring laser diffraction in the forward and side‐scatter directions. To ensure cell clusters were not mistakenly identified as single‐cells, each of the forward and side‐scatter measurements were plotted as peak versus integral signals. Electronic gating was used to exclude events from consideration that had a larger‐than‐normal forward‐ or side‐scatter signal. Fifteen plates were sorted for each NT sequence. Following plating, the single‐cell sorted plates were placed in a humidified incubator (36.0°C, 6% CO_2_).

The plates containing sorted cells were visually scored for outgrowth, and the CDCLs were transferred to deep‐well plates (DWPs) 14 days post‐sorting. After transfer of 80 colonies (TI) or 160 colonies (RI) per NT sequence to a DWP, all wells received CDCL Maintenance Medium. DWPs were sealed with Breathe‐Easy™ membranes (USA Scientific, Ocala, FL) and placed in a humidified incubator at 36.5°C with 6% CO_2_, static.

A shaken culture evaluation in the DWP format was performed. The plates were placed in Kuhner incubator, static, at 36°C, 6% CO_2_, and 85% relative humidity (RH) for 7 days. The vc number for each culture was estimated using the Cell Titer Blue (CTB) assay (Promega, Madison, WI), according to manufacturer's protocol. To increase the vc number for launch of the terminal study, each DWP was split into two identical DWPs by combining 100 μL of well‐mixed cell culture with 400 μL of Deep‐well Expression Medium. The two sets of plates were then started shaking at 325 rpm, 36°C, 6% CO_2_, and 85% RH and allowed to grow for 4 days. On the fourth day of shaking, expression study for the two sets of consolidated deep‐well plates was launched.

The terminal study was conducted in Kuhner incubators at 36°C, 325 rpm, 6% CO_2_, and 85% RH. On day 6, one of the sets of plates from each media was sterilely sampled for vc number using the CTB assay. The remaining set of plates were fed a 1× concentration of Lilly feed at 100 μL per well and returned to the incubator. The plates were fed an additional 1x concentration of Lilly feed on day 10 at 65 μL per well. The plates were harvested and sampled for Octet (ForteBio) on day 14. The plates were centrifuged at 500 rpm for 10 min and a 20 μL sample of the cell‐free supernatant was sampled for Octet.

The productivity results from the deep‐well plate expression study were used to choose a group of the top six CDCLs for each of the NT sequences per integration method. Scaled CDCLs were transferred to individual wells of a six‐well plate containing 3 mL of CDCL Maintenance Medium. For CDCL transfer, the content from the appropriate well from the deep‐well plate (total of approximately 0.2 mL) was transferred into the well in the six‐well plate. Four days following transfer to the six‐well plates, the cells were transferred to E125 flasks (static) containing 7 mL of CDCL Maintenance Medium. Following 3 days in the static shake‐flask, the cells were fed with 15 mL medium, and returned to the incubator, shaking at 125 rpm. Three days following the feed and initiation of the shaking, the cell count and viability was determined for each clone via Vi‐Cell. All clones had a viability >96%, with cell densities that ranged between 0.5 and 4.4 × 10^6^ vc/mL. A five‐vial research cell bank (RCB) was prepared for each CDCL. A portion of each shake‐flask culture was pelleted via centrifugation and the supernates decanted. Each cell pellet was suspended in Cryopreservation Medium to a concentration of 1 × 10^7^ vc/mL and 1 mL aliquoted into each vial. The vials were packaged into Styrofoam rack “sandwiches” and stored at −80°C.

The scaled cell lines were evaluated in a fed‐batch shake‐flask evaluation, as described previously for the selected bulk cultures. The final cell lines from the TI set were identified based on productivity per relative GCN. Ideally, the relative GCN (explained below) for targeted integration is 1. This led to a set of CDCLs with approximately the same GCN and equivalent productivities.

For the RI cells, two CDCLs from each NT sequence were chosen to move forward. The first CDCL was chosen based on the relative GCN matching that from the targeted integration CDCL, as closely as possible, regardless of productivity (CO‐1 B, CO‐2 B, and CO‐3 B). While there were CDCLs from NAT with a relative GCN around 1, none of them produced any antibody. The second CDCL to move forward was chosen based on productivity, regardless of relative GCN (CO‐1 A, CO‐2 A, and CO‐3 A). This represents the method of choice when choosing top CDCLs in clinical cell line generation processes. For RI cells, there are a set of seven CDCLs moving forward—two each from CO‐1, CO‐2, and CO‐3, and one from NAT. See Appendix S1 for more details on how cell lines were chosen for use in this study.

### 5.8 Relative GCN analysis

For each CDCL, the relative GCN for LC and HC was determined. Primers and probes specific to the *LC* and *HC* sequences of four nucleotide variants (NAT, CO‐1, CO‐2, and CO‐3) of *mAb1‐IgG4* were designed (Table S10). The CHO49 detects Chinese Hamster Alu‐like repeat sequences interspersed throughout the Chinese Hamster genome and is used as a normalizing assay for the determination of gene copies in Chinese Hamster Ovary (CHO) cell‐derived production cell lines. All qPCR assays used here utilize TaqMan® probe‐based chemistry, and QuantStudio™ 7 Flex Real‐Time PCR System (Thermo Fisher Scientific).

To characterize transduced cell lines, cell pellets were prepared at ~5 × 10^7^ viable cells per pellet. Total DNA from the pellets were extracted using Qiagen AllPrep DNA/RNA Mini Kit (cat. #80204) according to the manufacture's protocol. The concentrations and quality of the isolated DNA were evaluated using NanoDrop 1000 Spectrophotometer (Thermo Fisher Scientific). The DNA samples were subjected to the qPCR assays listed above (LC or HC and CHO49).

#### 5.8.1 qPCR assay conditions

All qPCR reactions were carried out in separate single wells of 384‐well reaction plates. TaqMan® probe and mixed primer stock concentrations were prepared at (40×) 10 and 36 μM concentrations respectively to give 250 and 900 nM PCR reaction concentrations, respectively. Each qPCR reaction well contained 5 μL of input sample DNA, 7.5 μL 2X TaqMan® PCR Master Mix (Thermo Fisher Scientific), 900 nM forward and reverse primer, 250 nM probe and 30 ng/μL Baker's Yeast tRNA (Sigma Part# R‐5636).

The 384‐well plates were analyzed using a QuantStudio™ 7 using the default settings. The thermal cycler conditions were 50°C for 2 min, 95°C for 10 min, then 40 cycles of 95°C for 15 s followed by 60°C for 1 min.

#### 5.8.2 qPCR assay standards and controls

The expression plasmid corresponding to each LC and HC nucleotide variant was used to make standard curves for the LC and HC assays. The standard curve was created using triplicates of 10‐fold serial dilutions of the purified plasmid representing the range from 5 to 5,000,000 copies per reaction. Specificity was already determined in the qualification of the qPCR assay.

CHOGSKO DNA was used to make the standard curves for the CHO49 qPCR assay. The standard curve was created using triplicates of 10‐fold serial dilutions of the CHO (genomic DNA) gDNA representing the range from 10 to 10,000,000 femtogram (fg) per reaction.

#### 5.8.3 qPCR assay control

A no‐template control (NTC), which contains all reagents and primer‐probes but without any DNA templates was used to monitor airborne contaminations.

#### 5.8.4 GCN calculation

The relative GCN of the *LC* and *HC* genes in CHO cells is determined by comparing the *C*_T_ (Threshold cycle) values obtained from the gDNA isolated from the test articles with the standard curves generated by the reference plasmid DNA carrying the target amplicons. The input gDNA quantity of the test articles is normalized by the CHO49 assay. Two concentrations of the gDNA isolated from the test articles, 0.25 and 0.05 ng/μL, were used in the testing. In each reaction 5 μL of sample was added, making the final amount per reaction 1.25 and 0.25 ng (approximately 200 and 40 cells, respectively). All DNA samples had three replicates in the qPCR assays. The GCN is given as copies per diploid CHO cell (equivalent to 6.2 pg input DNA). The final GCN per cell was determined as the mean from the two dilutions and expressed as a range of mean ± 2‐fold, based on the ±2‐fold accuracy of the qPCR technology.

### 5.9 Production scales—shake flask and 36L

To generate the material evaluated in the studies, all selected bulk cultures and CDCLs were expanded for a 14‐day fed‐batch terminal study in either 2 × 80 mL shake‐flasks (details are the same as described above, except for volume change) or in a 36L Bellco bioreactor. One milliliter of a frozen cryovial of CHO cells expressing a humanized IgG4 antibody was thawed at 37°C and used to inoculate 20 mL of a proprietary, chemically defined medium. The cell culture suspension was incubated in a non‐humidified incubator at 36°C, 6% CO_2_, with agitation at 125 rpm. The cell culture was expanded every 3 or 4 days to a total volume of approximately 3 L and used to inoculate a Bellco bioreactor containing approximately 30 L of the same chemically defined medium. Bioreactor growth conditions were at an initial temperature of 36°C, the pH controlled at 7.0 (±0.2), and dissolved oxygen controlled at 70%. Bioreactor temperature was reduced to 32°C on day 4. Chemically defined feeds were added to the reactor on days 4, 7, and 10 of incubation. The bioreactor was sampled daily to analyze cell growth with a Vi‐cell XR Cell Viability Analyzer (Beckman Coulter Diagnostics, Brea, CA). Metabolites, gases, and electrolytes were also analyzed daily using a BioProfile Flex Analyzer (Nova Biomedical, Waltham, MA). Glucose was added as required to maintain a concentration of greater than 20 mM. On the 14th day after inoculation, the bioreactor culture was harvested through a Pall depth filter followed by a 0.22 m polishing filter and concentrated approximately 10‐fold via a TFF column (10 kDa MWCO). Supernatants from the expression studies were submitted for protein A capture.

### 5.10 Preparative protein A chromatography

Clarified CHO‐derived material was loaded onto a mAbSelect PrA affinity column at 20–40 g of product/L of PrA resin. The resin was washed with a neutral pH Tris buffer (20 mM Tris, pH 7), followed by Tris buffer containing sodium chloride (20 mM Tris, 1 M NaCl, pH 7) then re‐equilibrated into Tris buffer with no sodium chloride (20 mM Tris, pH 7). The eluted product pool pH was adjusted to pH 5.0 using Tris Base resulting in formation of precipitation that was removed using 0.22 μm filtration. Protein concentrations were determined at 280 nm.

### 5.11 Analytical size exclusion chromatography

Relative aggregate percent was estimated by analytical size exclusion using an Agilent 1260 HPLC instrument and absorbance detected at 280 nm. Sample was loaded onto a TOSOH TSK gel G3000 SWxL (7.8 mm × 30 cm, 5 μm) column and operated at 0.50 mL/min using an isocratic gradient of 50 mM potassium phosphate, 350 mM NaCl, pH 7.0.

### 5.12 CE‐iCE, CE‐SDS, and glycosylation analysis

Process changes, such as codon optimization or manufacturing at different scales and/or switching the production process mode may impact co‐ and post‐translational protein modifications, including changes in the charge and/or glycosylation patterns and protein processing in the ER. To address changes in some of these attributes charge variants were determined using imaged capillary isoelectric focusing (CE‐iCE) as previously described.^37^ Capillary electrophoresis‐sodium dodecyl sulfate (CE‐SDS) was performed as previously described.^37^ Glycan profiles of all purified protein were further also determined as previously described.^37^

### 5.13 HRS analysis

The HRS assay was developed at Eli Lilly to measure carbonyl type oxidation in proteins and performed as previously described.^33^, ^37^

### 5.14 Gel electrophoresis

Protein concentrations were measured by NanoDrop One (Thermo Fisher Scientific). For nonreducing SDS‐PAGE, equal amounts of protein were prepared with lithium dodecyl sulfate (LDS) buffer (Thermo Fisher Scientific) and separated on NuPAGE 4%–12% bis‐tris midi gels in MOPS running buffer (Thermo Fisher Scientific). For NativePAGE, equal amounts of protein were prepared with Native Sample Buffer (Thermo Fisher Scientific) and separated on NativePAGE 3%–12% bis‐tris mini gels following the manufacturer's protocol (Thermo Fisher Scientific). NativePAGE gels were separated in either light blue cathode buffer (0.002% Coomassie G‐250) only or dark blue buffer (0.02% Coomassie G‐250) for 1/3 of the gel and light blue buffer for the remainder of the gel. Protein was visualized by silver staining of NuPAGE and NativePAGE gels following manufacturer's protocol (SilverQuest™, Thermo Fisher Scientific). Images of gels were obtained using iBright™ CL750 Imaging System (Thermo Fisher Scientific). All protein samples were analyzed by nonreducing SDS‐PAGE and NativePAGE at least three times.

### 5.15 Binding kinetics

Antibody samples were diluted to 50 μg/mL in HEPES buffered saline containing 0.05% Tween20 (10 mM HEPES, 150 mM NaCl, 3 mM EDTA, 0.05% Tween20, pH 7.4) for binding kinetic analysis on a Biacore 8 K+ surface plasmon resonance (SPR). For each cycle approximately 140 resonance units (RU) of antibody sample were captured by anti‐human IgG (Fc‐specific) which was previously immobilized to a CM5 chip via amine‐coupling. The antigen was subsequently injected over the chip at a flow rate of 30 μL/min for 180 s and allowed to dissociate from antibody samples for 300 s before regeneration of the anti‐IgG surface with 10 mM glycine‐HCl pH 2.0. For each antibody sample, seven different concentrations of antigen and a buffer blank were injected over captured antibodies at 25°C. Kinetic parameters were evaluated from the association and dissociation phases of the sensorgram. Data were blank‐subtracted and fit to a 1:1 binding model with Biacore Evaluation software. The equilibrium dissociation constants (*K*_d_) were determined by the software from dividing the *k*_off_ by the *k*_on_.

### 5.16 ELISA

A 96‐well immunosorbent microplate (Nunc™, Thermo Scientific) was coated with human antigen overnight at 2–8°C in DPBS (Gibco) and subsequently blocked with a casein solution in TBS (Thermo Scientific). Human mAbs with various synonymous mutations and manufactured via different cell culture methods, were added to the plate, resulting in an antigen‐mAb complex bound to the surface of the microplate. Bound complex was reacted with HRP conjugated anti‐human IgG (Jackson Laboratories) and measured spectrophotometrically (SpectraMax Microplate Reader) after adding the 3,3′,5,5′‐tetramethylbenzidine (TMB) substrate mixture (Thermo Scientific). The reaction was stopped with KPL TMB Stop Solution (Seracare). Each mAb was independently diluted to a top concentration of 36 μg/mL and serially diluted in casein buffer seven times and tested on three plates. The median absorbance was graphed as a function of the log concentration of mAb. The data is fit using a 4‐parameter logistic model and the relative potency of the sample compared to the reference standard (RI‐CDCL‐36L‐NAT) was calculated by SoftmaxPro using a constrained fit. Relative efficacy was calculated by comparing the absorbance at 450 nm at 36 μg/mL of each sample to the reference standard (RI‐CDCL‐36L‐NAT) on each plate.

### 5.17 Statistical analysis

Statistical analysis was performed among groups that did not have obvious subpopulations. If variances were visually consistent, an ordinary one‐way analysis of variance (ANOVA) with Tukey's multiple comparisons test was used to compare means in data comparing the four NT sequences, a two‐way ANOVA with Tukey's multiple comparisons test was used to compare means among data from multiple variables, and an ordinary one‐way ANOVA with Dunnett's multiple comparisons test was used to compare means of samples against the reference sample (in ELISA relative response data). If variances were not visually consistent, Welch's *t*‐test (two‐tailed) was performed. Significant differences between groups that differ in one variable are indicated on graphs: *p < 0.05, **p < 0.01, ***p < 0.001, ****p < 0.0001. Data are presented as mean ± standard deviation (SD). All statistical analyses were performed using GraphPad Prism (version 9.5.1). Details for all results from statistical testing can be found in Appendix S2.

## AUTHOR CONTRIBUTIONS

**Nayiri M. Kaissarian:** Investigation; formal analysis; writing – original draft; writing – review and editing; visualization. **Stephanie L. Sandefur:** Investigation; writing – review and editing. **Arnab Ghosh:** Investigation. **Upendra K. Katneni:** Writing – review and editing; visualization. **Wendy Walton:** Investigation; writing – review and editing. **Christopher C. Frye:** Conceptualization; methodology; resources; supervision; funding acquisition; writing – review and editing. **Anton A. Komar:** Conceptualization; methodology; resources; supervision; funding acquisition; writing – review and editing. **Chava Kimchi‐Sarfaty:** Conceptualization; methodology; resources; supervision; funding acquisition; writing – review and editing.

## CONFLICT OF INTEREST STATEMENT

Anton A. Komar is a founder of SATOR Therapeutics LLC. Anton A. Komar's spouse is a founder of DAPCEL, Inc. and SATOR Therapeutics LLC. The other authors declare no competing interests.

## Supporting information


**APPENDIX S1:** Supplementary note.

**APPENDIX S2:** Extended statistics.

**FIGURE S1:** Relative synonymous codon usage (RSCU) in the NAT and CO‐1, 2, and 3 (a) heavy chain (HC) and (b) light chain (LC) mAb1‐IgG4 sequences. Heatmaps indicate the RSCU for all codons among the four (a) HC and (b) LC sequences. Darker blue colors indicate higher RSCU values. Lighter blue colors indicate lower RSCU values.
**FIGURE S2.** Relative synonymous codon pair usage (RSCPU) in the NAT and CO‐1, 2, and 3 (a) heavy chain (HC) and (b) light chain (LC) mAb1‐IgG4 sequences. Heatmaps indicate RSCPU values for all codon pairs present in the HC and LC sequences. The heatmaps are broken into multiple parts (a, HC‐8 and b, LC‐5) to be legible. Codon pairs are in alphabetical order. Legend indicates RSPCU values for each codon pair present in these sequences. Darker gray/black colors indicate higher RSPCU values. Lighter gray colors indicate lower RSCPU values.
**FIGURE S3.** The three CO HC and LC sequences were aligned with the native sequence using MegAlign Pro. This figure shows differences from the NAT sequence. Nucleotides are color coded: A—red; T—green; C—blue; G—yellow.
**FIGURE S4.** Binding kinetics. (a) Although statistically significant (Welch's *t*‐test, *p* = 0.0274), the variation in binding affinity between random integration (RI) and targeted integration (TI) groups is within method variability. (b) Kinetic rate constants, *k*_a_ and *k*_d_, are highly correlated indicating that faster on rate (*k*_a_) corresponds to faster off rate (*k*_d_). This is common with mAb–antigen interactions. (c) The interaction effect between *k*_a_ and relative potency (from ELISA) is observed for the RI‐Bulk‐SF group (right) but not for TI‐Bulk‐SF (left) on correlation plots (see also Figures 6b and S6 for ELISA data).
**FIGURE S5.** Circular dichroism (CD) spectra obtained at 5°C. Far‐UV spectra—each spectrum is the average of three spectra obtained for each sample. There are no observable differences among circular dichroism (CD) spectra. (a) Molar residue ellipticity (MRE) and (b) each spectrum normalized to highest MRE per sample.
**FIGURE S6.** ELISA dose response curves. Each ELISA setup allowed for triplicate measurement of one reference sample (#16) and three test samples. Each graph in this figure is representative of the three repeat ELISA plates measured for each set of three test samples. Compared to the reference sample, eight samples had higher upper asymptotes (#1–4 and #31–34). This observation is also reflected in the relative response at the highest dose reported in Figure 6a.

**TABLE S1:** CE‐SDS non‐reduced purity, fragments, aggregates.
**TABLE S2:** CE‐SDS reduced purity, fragments aggregates, % non‐glycosylated HC.
**TABLE S3:** SEC monomer, total fragments, total aggregates.
**TABLE S4:** CE‐iCE main peak, acidic variants, basic variants.
**TABLE S5:** Glycosylation—G0, G0F, G0F‐GlcNAc, G1F.
**TABLE S6:** Glycosylation—G1F*, G1Fs, G2F, Man‐5.
**TABLE S7:** Relative gene copy number (GCN), hydroxylamine species (HRS).
**TABLE S8:** Titer, protein A capture, specific productivity.
**TABLE S9:** KD, relative response at 36 μg/mL, relative potency.

**TABLE S10.** Primer‐probe sequences of the qPCR assays.

## Data Availability

The data that support the findings of this study are available from the corresponding author upon reasonable request.
