# Supplementary material for: Investigating the impact of synonymous gene recoding on a recombinantly expressed monoclonal antibody under different process parameters
Source: Bioeng Transl Med. 2025 Jan 27;10(3):e10750. doi: 10.1002/btm2.10750 (PMC12079346; doi:10.1002/btm2.10750)
Supplement: Supplementary file 1 — APPENDIX S1: Supplementary note. [file BTM2-10-e10750-s001.docx]

**Supplementary Note**

**Generation of Bulk cultures and Clonally Derived Cell Lines (CDCLs)**

Following selection using GS and G418, there were no observable differences in recovery among TI cells (Fig. 1, left), but there was a noticeable difference among RI cells depending on the nucleotide (NT) sequence. The NAT bulk culture dropped to a much lower viability through selection than did the CO sequences. All three CO sequences showed similar recovery curves (Fig. 1, right).

Selected bulk cultures were single cell sorted via FACS, and outgrowth was measured. There was an observable difference in percent outgrowth between the TI-NAT sequence compared to the TI-CO sequences. TI-CO-3 showed the highest outgrowth (Fig. 2, left). Contrary to the results from TI, the RI-NAT sequence showed the highest outgrowth percentage, and CO-2 had the lowest outgrowth (Fig. 2, right).

After transfer of 80 TI-CDCLs and 160 RI-CDCLs of each NT sequence into deep-well plates, CDCLs were screened for productivity. Although there was growth in 316 out of 320 wells from TI, there were differences in the number of CDCLs that produced measurable titer (Fig. 3, left). The TI-CO-1 plate contained only 25 out of 80 wells with measurable titer, while NAT, CO-2, and CO-3 plates had 68, 61, and 66, respectively, out of 80 wells. Even though the CO-1 group had the fewest wells showing measurable titer, it had the highest mean, and contained the cell line with the highest titer. Similar to TI, although there was growth in 434 out of 640 wells from RI, there was a difference in the number of wells containing CDCLs that produced measurable titer (Fig. 3, right). The NAT set had only four wells with measurable titer, while CO-1, CO-2, and CO-3 had 93, 92, and 95 wells, respectively, out of 160 wells. All NT sequences showed equivalent mean titers.

Six TI-CDCLs from each NT sequence were scaled up and analyzed in a shake-flask fed-batch terminal study (Fig. 4). The top CDCL from each NT sequence was chosen based on titer per relative gene copy number (GCN) for the LC and HC (Fig. 4). These four cell lines were used to produce the protein for the TI-CDCLs used in this study in both the shake flask and 36L production scales. In the study, the productivities were very similar. For the shake-flasks, the productivities ranged from 0.89 – 1.13 g/L and the 36L productivities ranged from 0.81 – 0.93 g/L (Fig. 5).

Six RI-CDCLs from each NT sequence (four with the highest productivity in the small-scale expression study, and two with relative GCN similar to the TI CDCLs) were scaled up and analyzed in a shake-flask fed-batch terminal study (Fig. 4). The productivity was measured using the BioHT. Two RI-CDCLs were chosen from each group for further evaluation. Even though integration in this part is random, CDCLs were chosen that contained a relative GCN similar to the TI-CDCLs, regardless of productivity. The exception to this was the NAT set. There were no CDCLs with an equivalent GCN to the TI-CDCL that had measurable titer (>0.06 g/L). The second CDCL chosen was based on productivity, regardless of the relative GCN. When these seven CDCLs were analyzed in the larger-scale studies, the productivities were slightly variable, depending on the relative GCN. However, there was correlation between the productivity scales (Fig. 5).

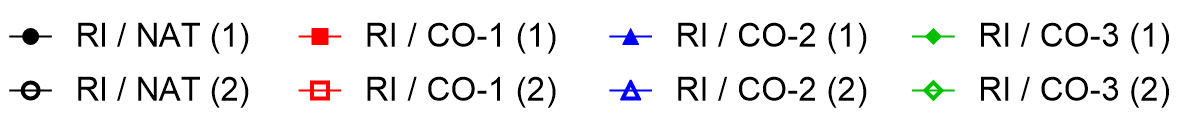


**Figure 1**. Recovery after selection with the different NT sequences in TI (GS and G418 selection, left) and RI (GS selection, right). Total viable cell number (top) and viability (bottom). For TI, GS selection began on day 4, and G418 selection began on day 18. For RI, GS selection began on day 1 post-electroporation.

**Figure 2**. Percent of wells with at least 15% confluency after FACS sorting.

|  | TI | | | | RI | | | |
| --- | --- | --- | --- | --- | --- | --- | --- | --- |
|  | NAT | CO-1 | CO-2 | CO-3 | NAT | CO-1 | CO-2 | CO-3 |
| Number of values | 68 | 25 | 61 | 66 | 4 | 93 | 92 | 95 |

**Figure 3**. Average titer in CDCL wells on Day 14 of the primary ACES terminal study evaluation (see online methods section). TI started with 80 wells, and RI started with 160 wells. Wells with productivity ≤ 0.06 g/L were excluded.

**Figure 4**. Relative gene copy number (rGCN) and productivity from the TI (top) and RI (bottom) CDCLs. The squares represent the rGCN for heavy chain (HC, blue) and light chain (LC, red), with the circles representing the productivity (black circles). Titer was measured on day 14 for TI and day 10 for RI.

**Figure 5**. Relative gene copy number (rGCN) and titer of RI (panels A and B) TI (panels C and D) CDCLs chosen for use in this study. The squares represent the rGCN for heavy chain (HC, blue) and light chain (LC, red), and the circles represent the productivity (black circles). Panels A and C include the shake flask (SF) samples. Panels B and D include the 36L samples.
