# Supplementary material for: Investigating the impact of synonymous gene recoding on a recombinantly expressed monoclonal antibody under different process parameters
Source: Bioeng Transl Med. 2025 Jan 27;10(3):e10750. doi: 10.1002/btm2.10750 (PMC12079346; doi:10.1002/btm2.10750)
Supplement: Supplementary file 3 — FIGURE S1: Relative synonymous codon usage (RSCU) in the NAT and CO‐1, 2, and 3 (a) heavy chain (HC) and (b) light chain (LC) mAb1‐IgG4 sequences. Heatmaps indicate the RSCU for all codons among the four (a) HC and (b) LC sequences. Darker blue colors indicate higher RSCU values. Lighter blue colors indicate lower RSCU values. FIGURE S2. Relative synonymous codon pair usage (RSCPU) in the NAT and CO‐1, 2, and 3 (a) heavy chain (HC) and (b) light chain (LC) mAb1‐IgG4 sequences. Heatmaps indicate RSCPU values for all codon pairs present in the HC and LC sequences. The heatmaps are broken into multiple parts (a, HC‐8 and b, LC‐5) to be legible. Codon pairs are in alphabetical order. Legend indicates RSPCU values for each codon pair present in these sequences. Darker gray/black colors indicate higher RSPCU values. Lighter gray colors indicate lower RSCPU values. FIGURE S3. The three CO HC and LC sequences were aligned with the native sequence using MegAlign Pro. This figure shows differences from the NAT sequence. Nucleotides are color coded: A—red; T—green; C—blue; G—yellow. FIGURE S4. Binding kinetics. (a) Although statistically significant (Welch's t‐test, p = 0.0274), the variation in binding affinity between random integration (RI) and targeted integration (TI) groups is within method variability. (b) Kinetic rate constants, k a and k d, are highly correlated indicating that faster on rate (k a) corresponds to faster off rate (k d). This is common with mAb–antigen interactions. (c) The interaction effect between k a and relative potency (from ELISA) is observed for the RI‐Bulk‐SF group (right) but not for TI‐Bulk‐SF (left) on correlation plots (see also Figures 6b and S6 for ELISA data). FIGURE S5. Circular dichroism (CD) spectra obtained at 5°C. Far‐UV spectra—each spectrum is the average of three spectra obtained for each sample. There are no observable differences among circular dichroism (CD) spectra. (a) Molar residue ellipticity (MRE) and (b) each spectrum no [file BTM2-10-e10750-s005.pdf]

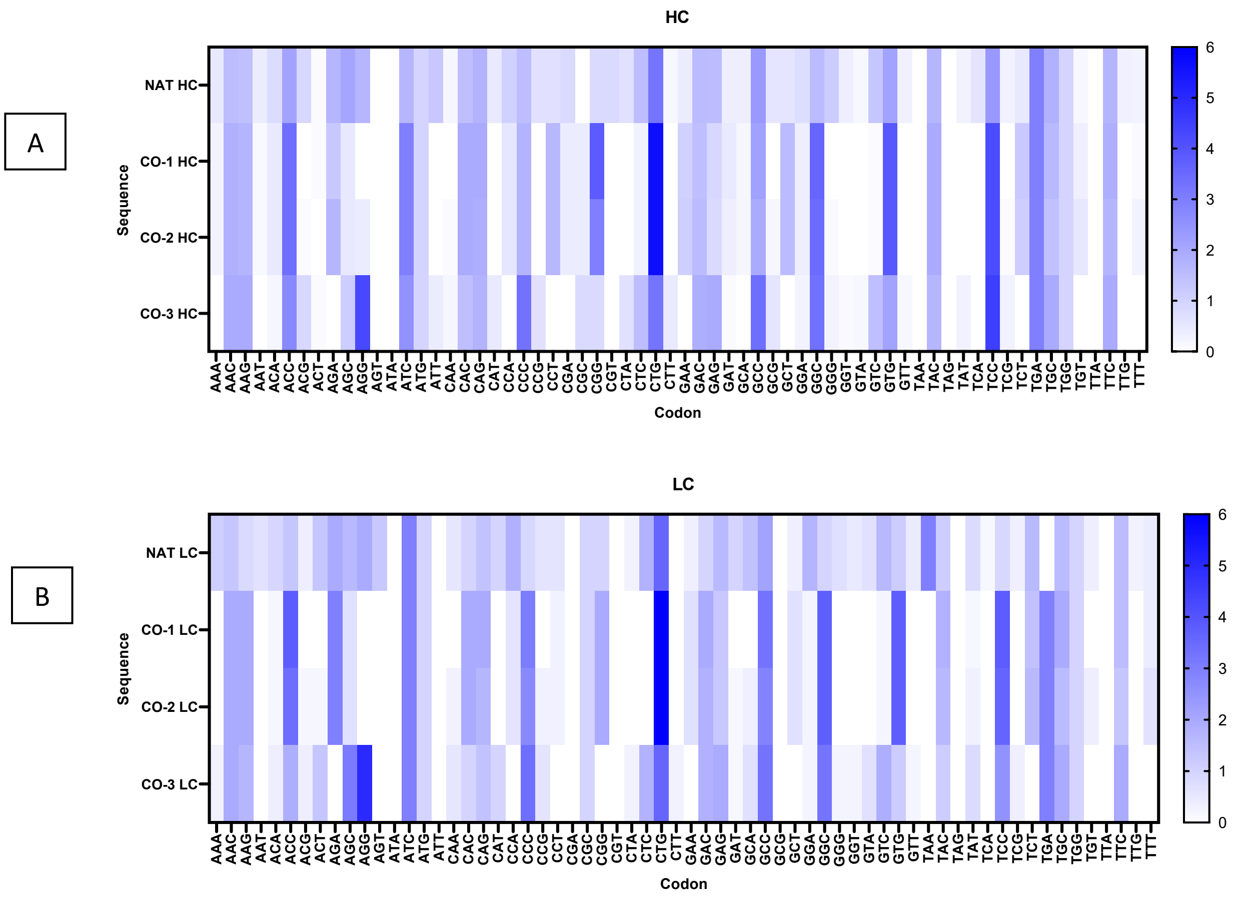

Supplementary Figure S1. Relative synonymous codon usage (RSCU) in the *NAT* and *CO-1*, *2*, and *3* (**A**) *heavy chain* (*HC*) and (**B**) *light chain* (*LC*) *mAb1-IgG4* sequences. Heatmaps indicate the RSCU for all codons among the four (**A**) *HC* and (**B**) *LC* sequences. Darker blue colors indicate higher RSCU values. Lighter blue colors indicate lower RSCU values.

A

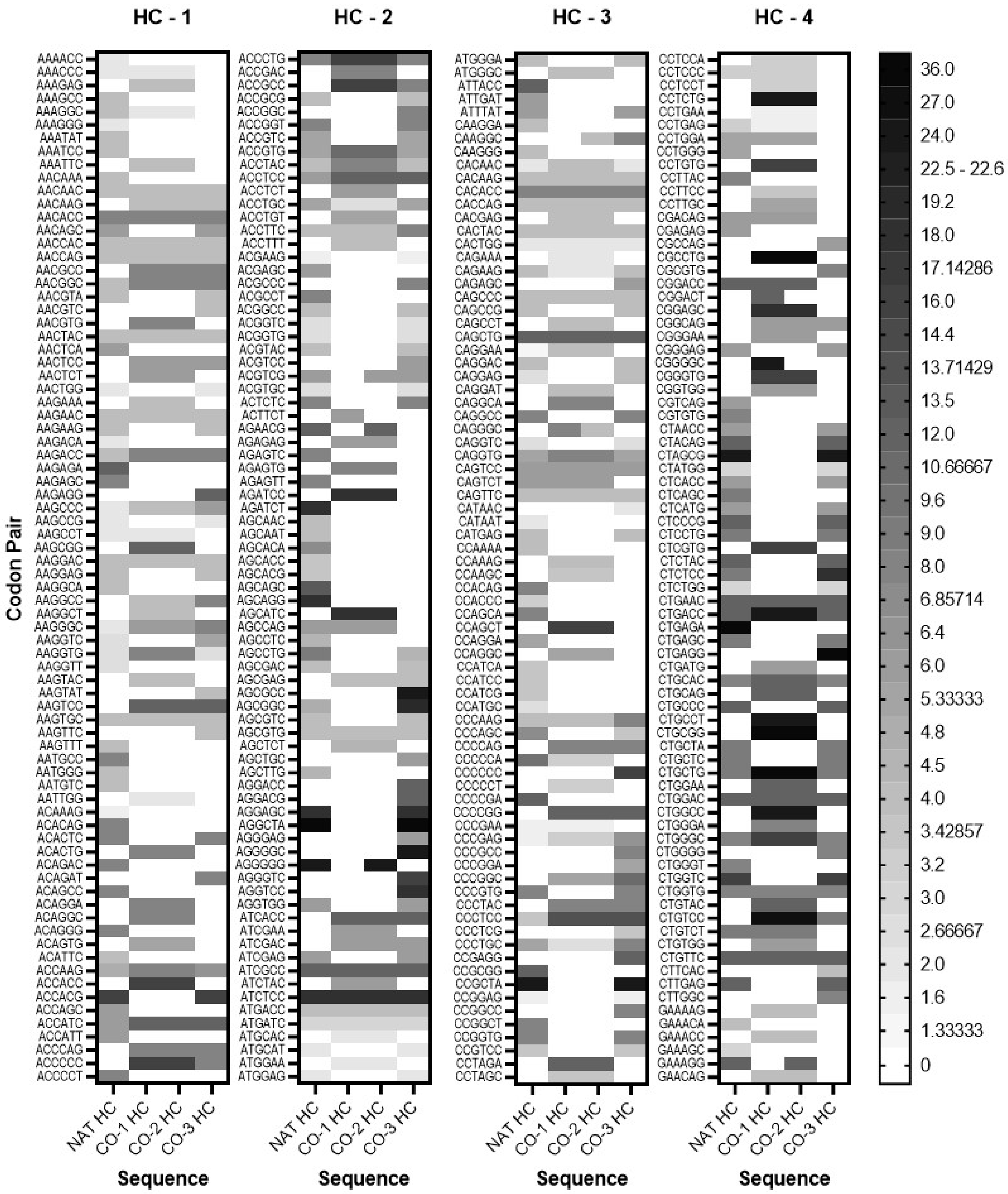
HC - 1
HC - 2
HC - 3
HC - 4
Codon Pair
NAT HC
CO-1 HC
CO-2 HC
CO-3 HC
Sequence
36.0
27.0
24.0
22.5 - 22.6
19.2
18.0
17.14286
16.0
14.4
13.71429
13.5
12.0
10.66667
9.6
9.0
8.0
6.85714
6.4
6.0
5.33333
4.8
4.5
4.0
3.42857
3.2
3.0
2.66667
2.0
1.6
1.33333
0

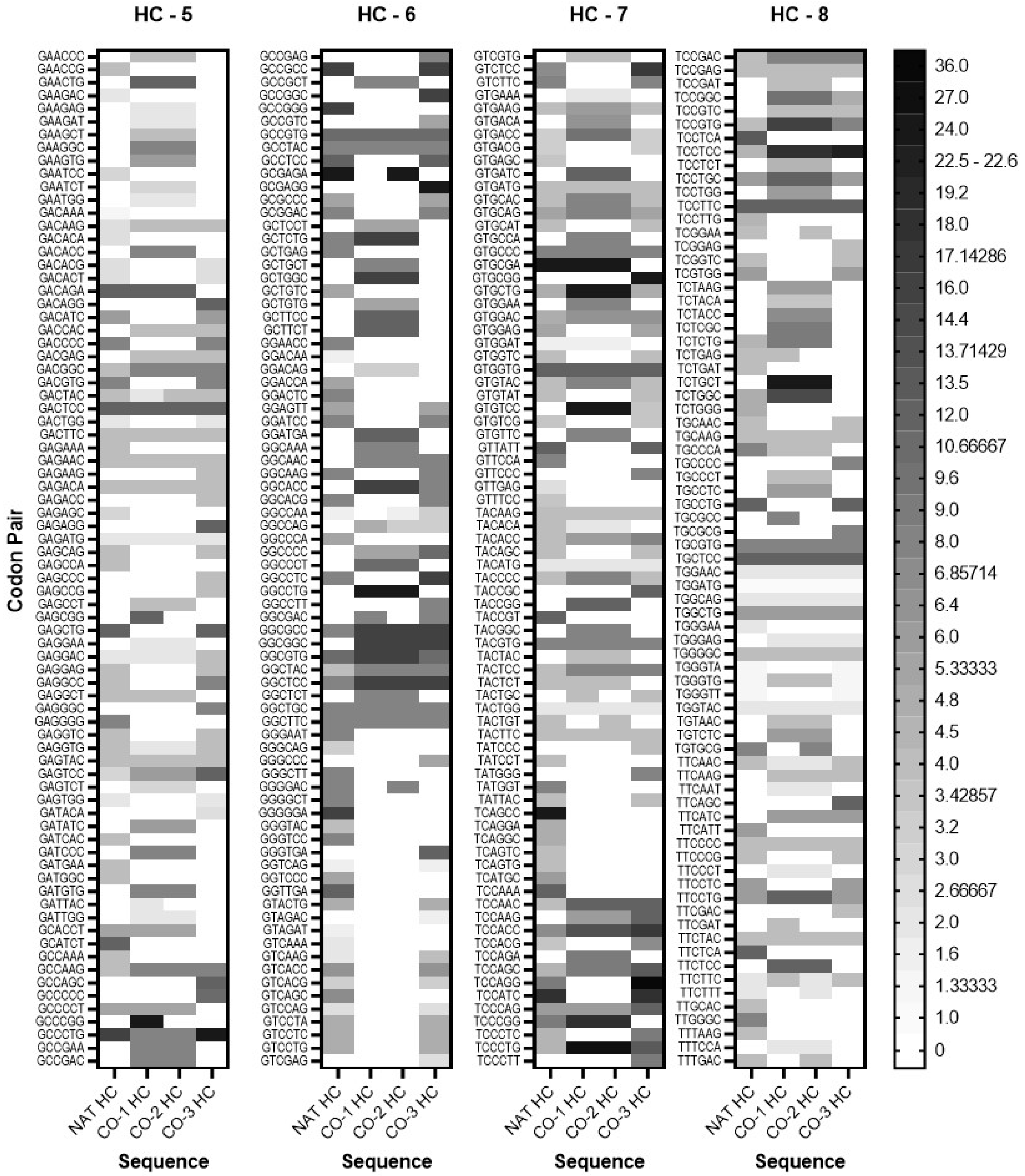
HC - 5
HC - 6
HC - 7
HC - 8
Codon Pair
NAT HC
CO-1 HC
CO-2 HC
CO-3 HC
Sequence
36.0
27.0
24.0
22.5 - 22.6
19.2
18.0
17.14286
16.0
14.4
13.71429
13.5
12.0
10.66667
9.6
9.0
8.0
6.85714
6.4
6.0
5.33333
4.8
4.5
4.0
3.42857
3.2
3.0
2.66667
2.0
1.6
1.33333
1.0
0

B

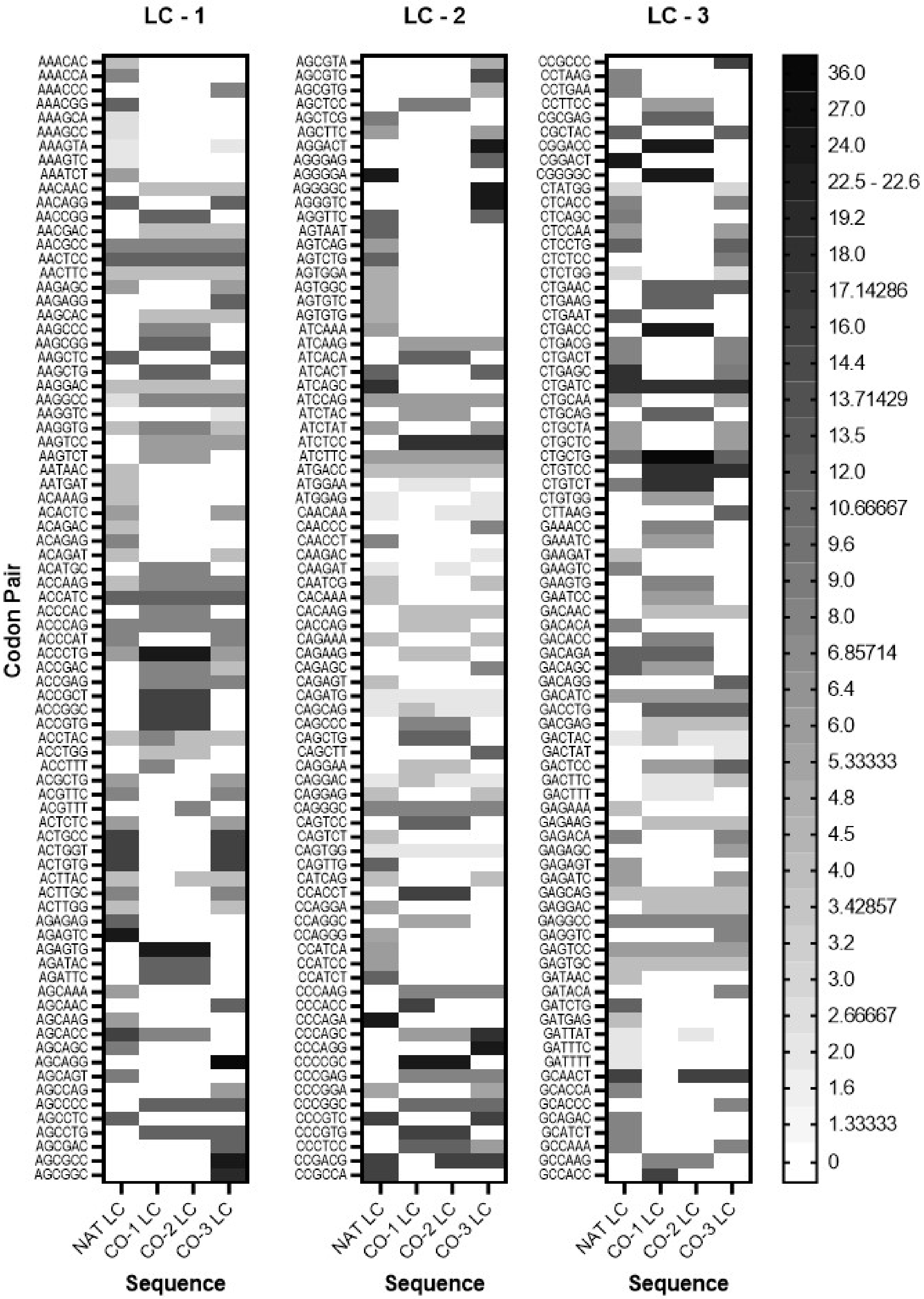
LC - 1
LC - 2
LC - 3
Codon Pair
Sequence
NAT LC
CO-1 LC
CO-2 LC
CO-3 LC
36.0
27.0
24.0
22.5 - 22.6
19.2
18.0
17.14286
16.0
14.4
13.71429
13.5
12.0
10.66667
9.6
9.0
8.0
6.85714
6.4
6.0
5.33333
4.8
4.5
4.0
3.42857
3.2
3.0
2.66667
2.0
1.6
1.33333
0

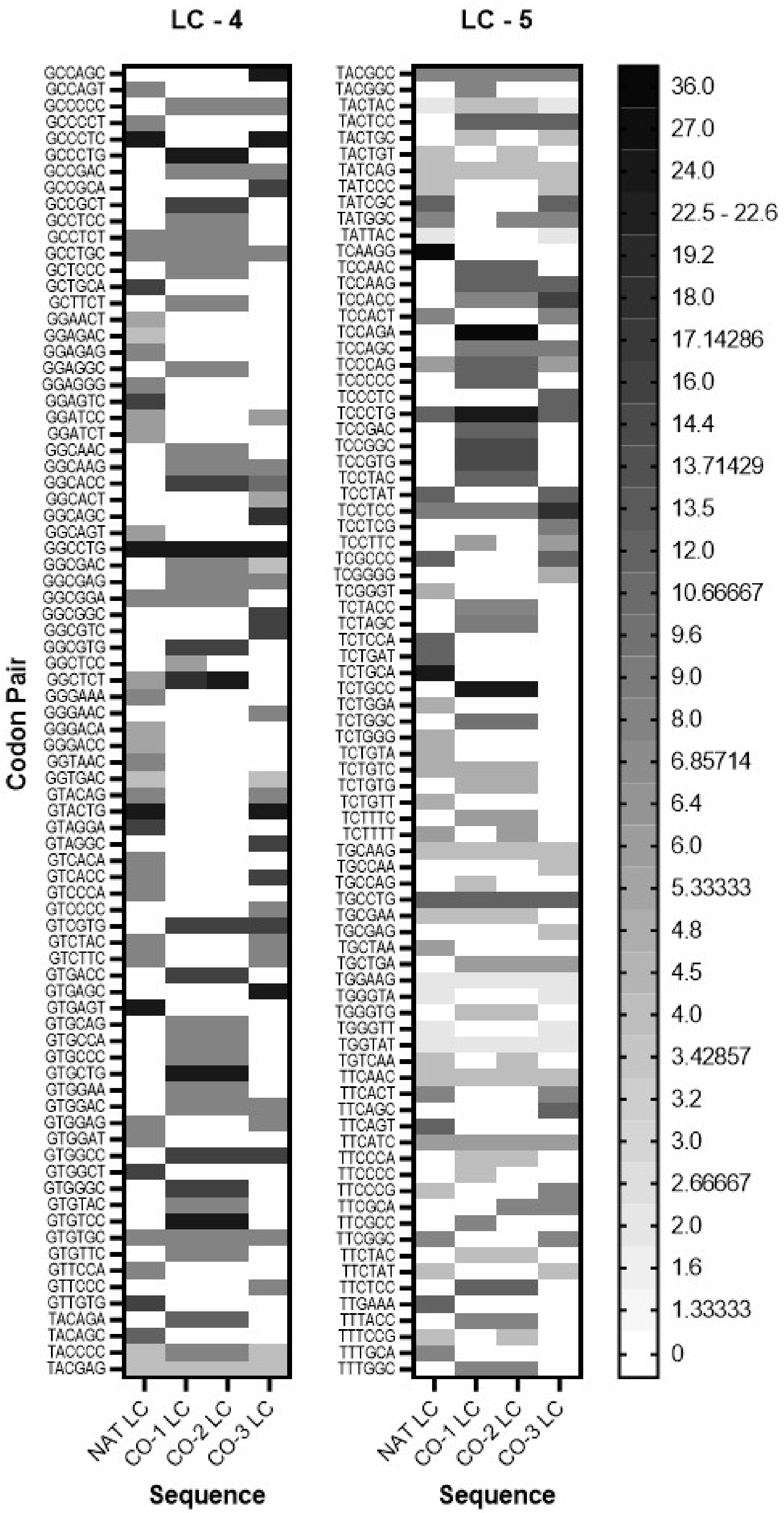
LC - 4
LC - 5
Codon Pair
GCCAGC
GCCAGT
GCCCCC
GCCCCT
GCCCTC
GCCCTG
GCCGAC
GCCGCA
GCCGCT
GCCTCC
GCCTCT
GCCTGC
GCTCCC
GCTGCA
GCTTCT
GGAACT
GGAGAC
GGAGAG
GGAGGC
GGAGGG
GGAGTC
GGATCC
GGATCT
GGCAAC
GGCAAG
GGCACC
GGCACT
GGCAGC
GGCAGT
GGCCTG
GGCGAC
GGCGAG
GGCGGA
GGCGGC
GGCGTC
GGCGTG
GGCTCC
GGCTCT
GGGAAA
GGGAAC
GGGACA
GGGACC
GGTAAC
GGTGAC
GTACAG
GTACTG
GTAGGA
GTAGGC
GTCACA
GTCACC
GTCCCA
GTCCCC
GTCGTG
GTCTAC
GTCTTC
GTGACC
GTGAGC
GTGAGT
GTGCAG
GTGCCA
GTGCCC
GTGCTG
GTGGAA
GTGGAC
GTGGAG
GTGGAT
GTGGCC
GTGGCT
GTGGGC
GTGTAC
GTGTCC
GTGTGC
GTGTTC
GTTCCA
GTTCCC
GTTGTG
TACAGA
TACAGC
TACCCC
TACGAG
NAT LC
CO-1 LC
CO-2 LC
CO-3 LC
Sequence
TACGCC
TACGGC
TACTAC
TACTCC
TACTGC
TACTGT
TATCAG
TATCCC
TATCGC
TATGGC
TATTAC
TCAAGG
TCCAAC
TCCAAG
TCCACC
TCCACT
TCCAGA
TCCAGC
TCCCAG
TCCCCC
TCCCTC
TCCCTG
TCCGAC
TCCGGC
TCCGTG
TCCTAC
TCCTAT
TCCTCC
TCCTCG
TCCTTC
TCGCCC
TCGGGG
TCGGGT
TCTACC
TCTAGC
TCTCCA
TCTGAT
TCTGCA
TCTGCC
TCTGGA
TCTGGC
TCTGGG
TCTGTA
TCTGTC
TCTGTG
TCTGTT
TCTTTC
TCTTTT
TGCAAG
TGCCAA
TGCCAG
TGCCTG
TGCGAA
TGCGAG
TGCTAA
TGCTGA
TGGAAG
TGGGTA
TGGGTG
TGGGTT
TGGTAT
TGTCAA
TTCAAC
TTCACT
TTCAGC
TTCAGT
TTCATC
TTCCCA
TTCCCC
TTCCCG
TTCGCA
TTCGCC
TTCGGC
TTCTAC
TTCTAT
TTCTCC
TTGAAA
TTTACC
TTTCCG
TTTGCA
TTTGGC
NAT LC
CO-1 LC
CO-2 LC
CO-3 LC
Sequence
36.0
27.0
24.0
22.5 - 22.6
19.2
18.0
17.14286
16.0
14.4
13.71429
13.5
12.0
10.66667
9.6
9.0
8.0
6.85714
6.4
6.0
5.33333
4.8
4.5
4.0
3.42857
3.2
3.0
2.66667
2.0
1.6
1.33333
0

Supplementary Figure S2. Relative synonymous codon pair usage (RSCPU) in the *NAT* and *CO-1*, *2*, and *3* (**A**) *heavy chain* (*HC*) and (**B**) *light chain* (*LC*) *mAb1-IgG4* sequences. Heatmaps indicate RSCPU values for all codon pairs present in the *HC* and *LC* sequences. The heatmaps are broken into multiple parts (**A.** HC-8 and **B.** LC-5) to be legible. Codon pairs are in alphabetical order. Legend indicates RSPCU values for each codon pair present in these sequences. Darker gray/black colors indicate higher RSPCU values. Lighter gray colors indicate lower RSCPU values.

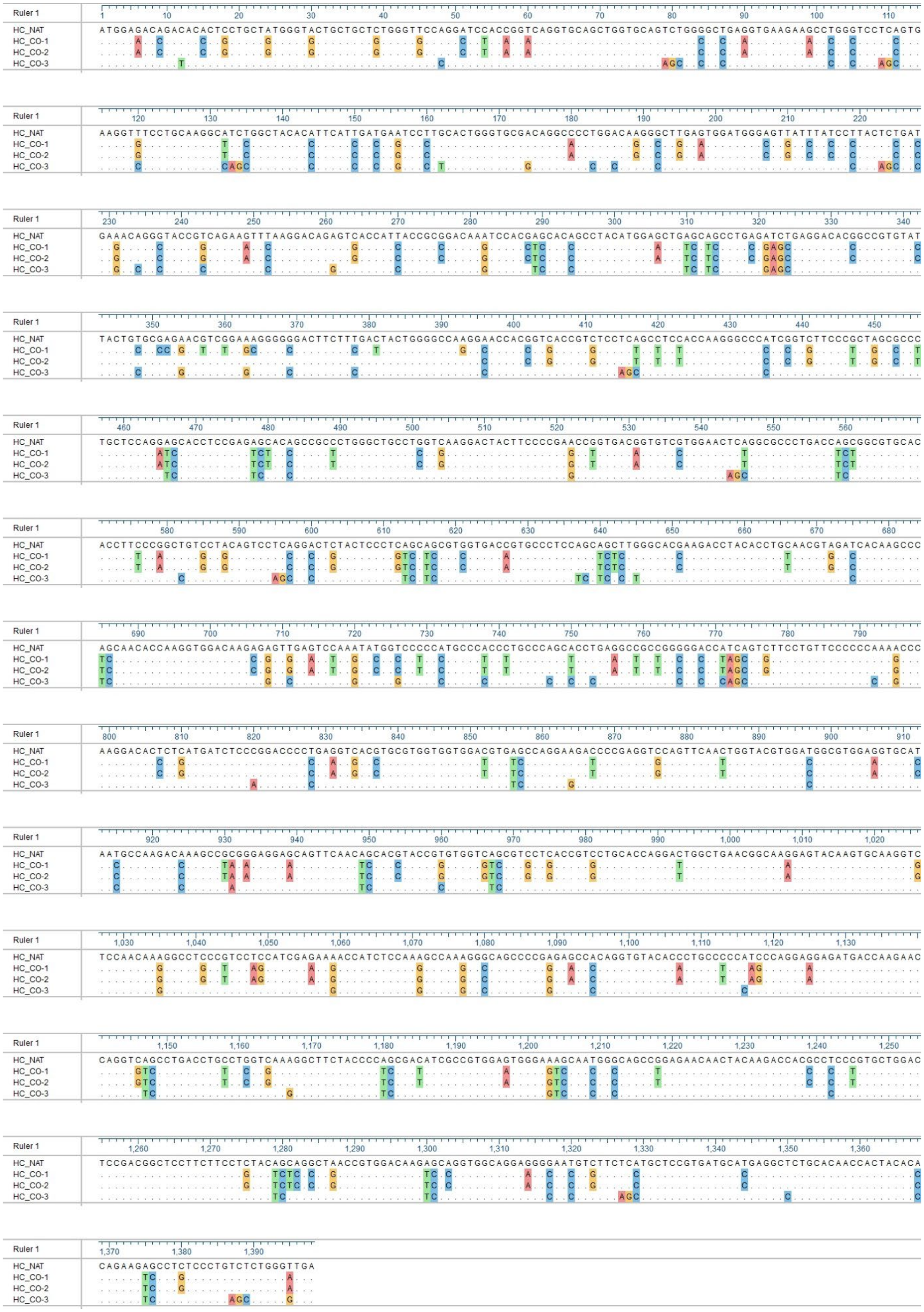
Ruler 1
HC_NAT
HC_CO-1
HC_CO-2
HC_CO-3

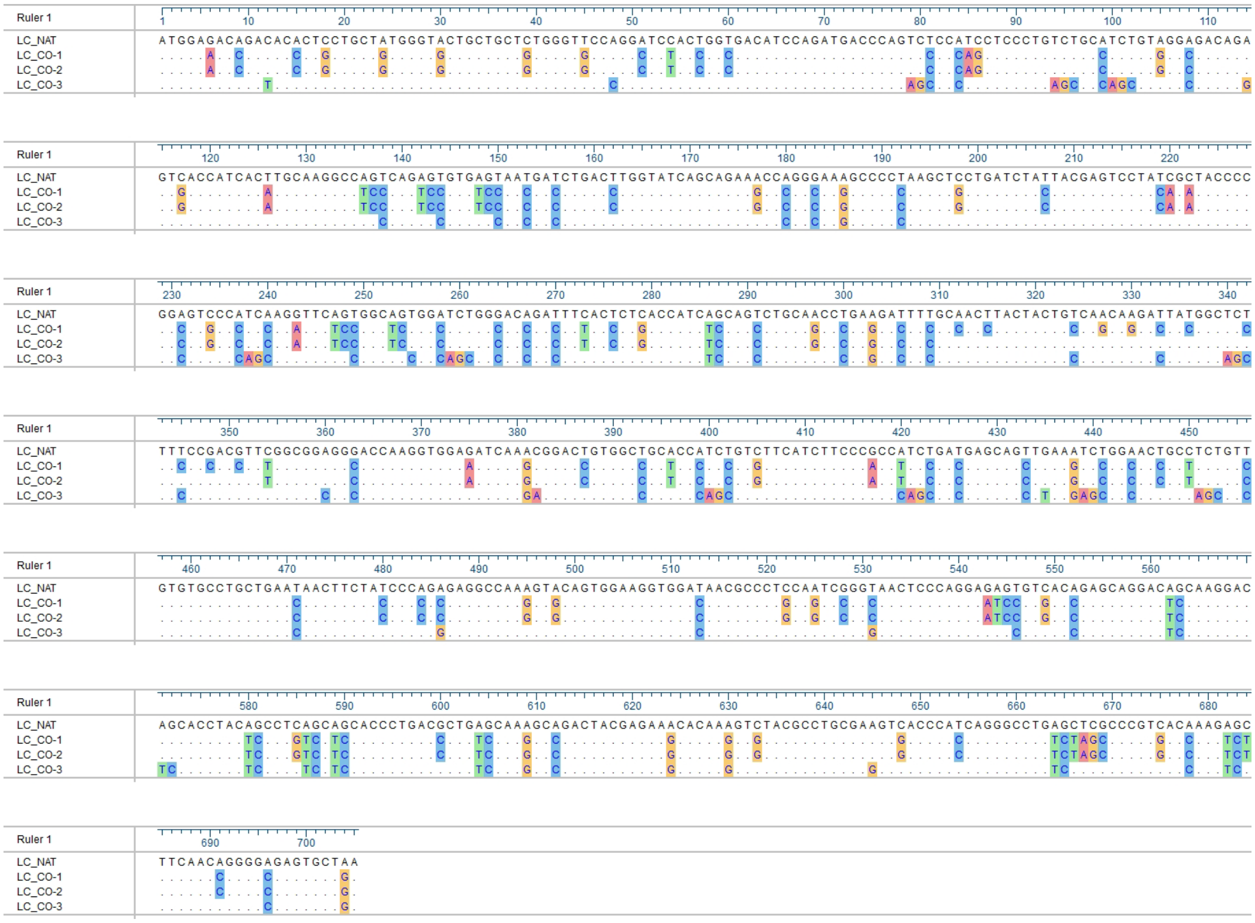

Supplementary Figure S3 The 3 CO HC and LC sequences were aligned with the native sequence using MegAlign Pro. This figure shows differences from the NAT sequence. Nucleotides are color coded: A red, T green, C blue, G yellow.

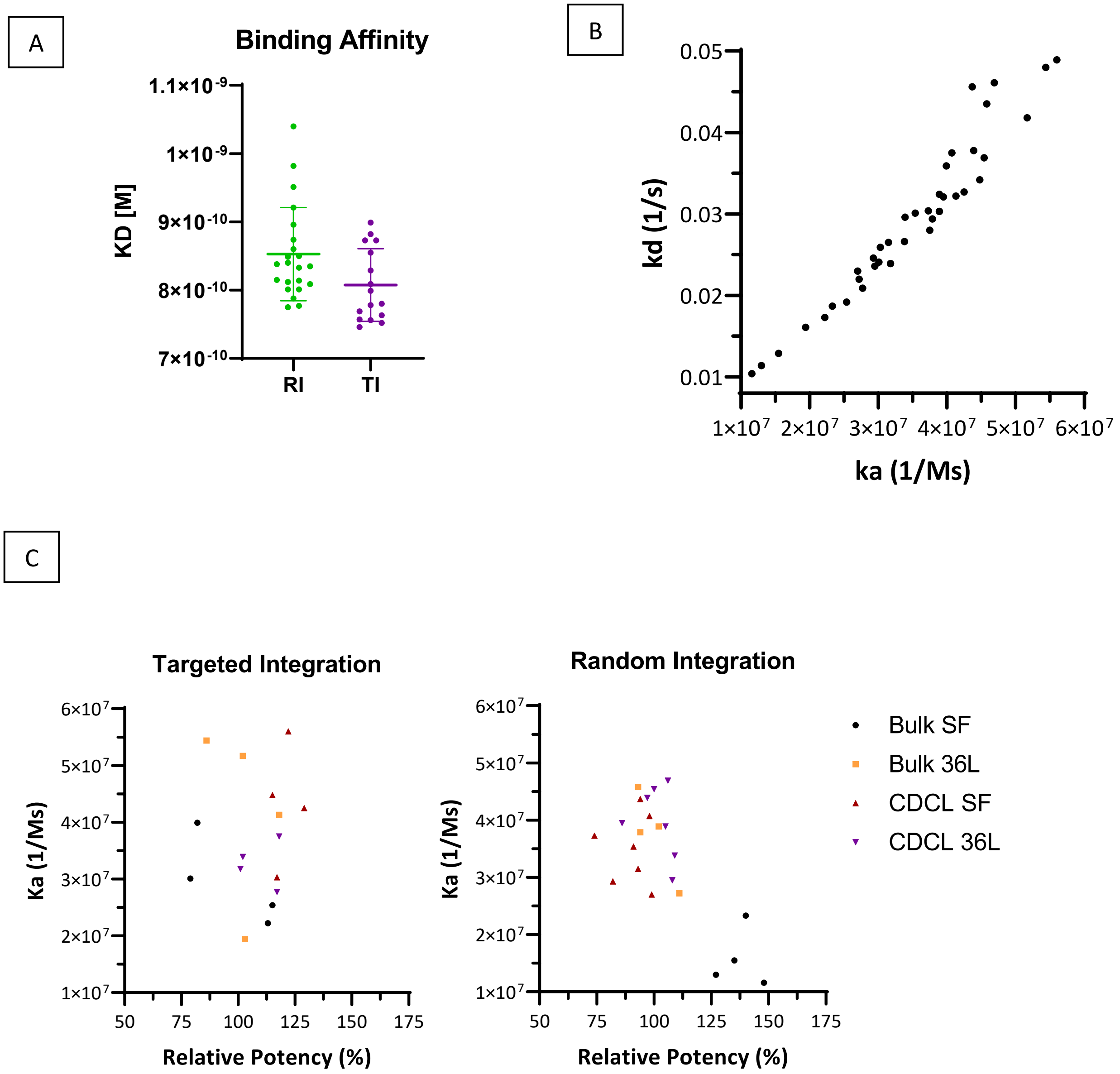

Supplementary Figure S4. Binding kinetics.

(A) Although statistically significant (Welch's t-test, $p=0.0274$), the variation in binding affinity between random integration (RI) and targeted integration (TI) groups is within method variability. **(B)** Kinetic rate constants, ka and kd, are highly correlated indicating that faster on rate (ka) corresponds to faster off rate (kd). This is common with mAb-antigen interactions. **(C)** The interaction effect between ka and relative potency (from ELISA) is observed for the RI-Bulk-SF group (right) but not for TI-Bulk-SF (left) on correlation plots (see also Fig. 6B and supplementary Fig. S6 for ELISA data).

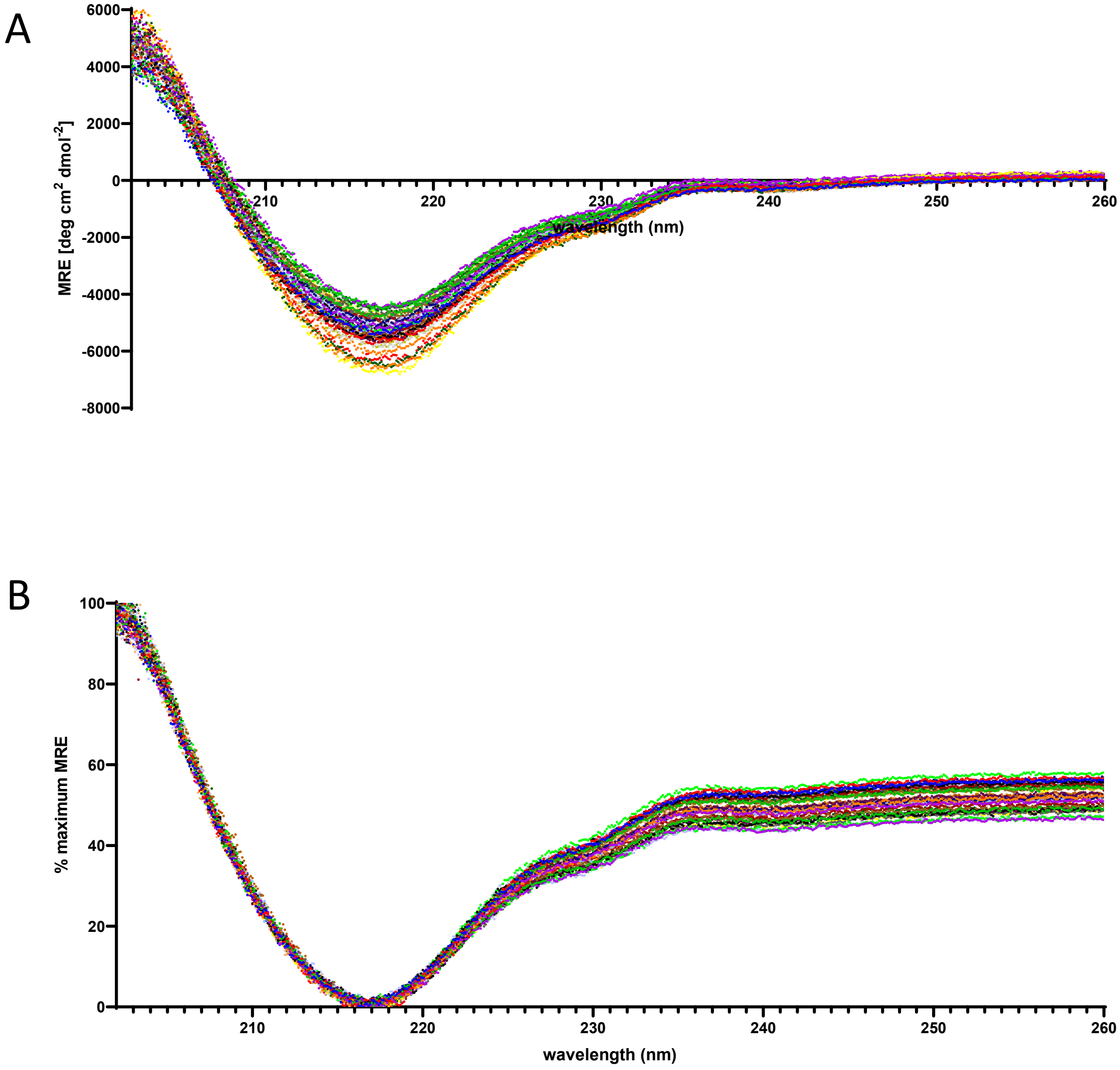

Supplementary Figure S5 Circular dichroism (CD) spectra obtained at 5 °C

Far-UV spectra - Each spectrum is the average of three spectra obtained for each sample. There are no observable differences among circular dichroism (CD) spectra. **A**. Molar residue ellipticity (MRE), **B**. Each spectrum normalized to highest MRE per sample.

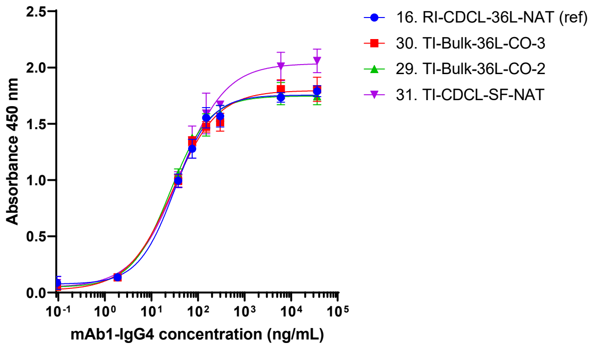
Absorbance 450 nm
mAb1-IgG4 concentration (ng/mL)
16. RI-CDCL-36L-NAT (ref)
30. TI-Bulk-36L-CO-3
29. TI-Bulk-36L-CO-2
31. TI-CDCL-SF-NAT

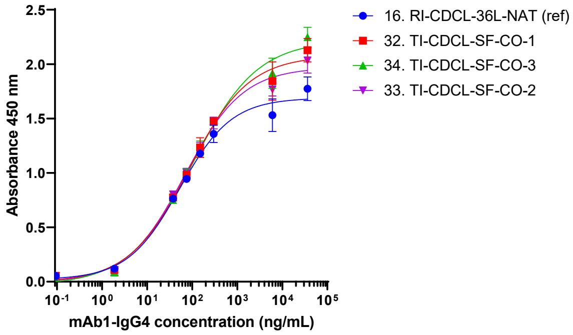
Absorbance 450 nm
mAb1-IgG4 concentration (ng/mL)
16. RI-CDCL-36L-NAT (ref)
32. TI-CDCL-SF-CO-1
34. TI-CDCL-SF-CO-3
33. TI-CDCL-SF-CO-2

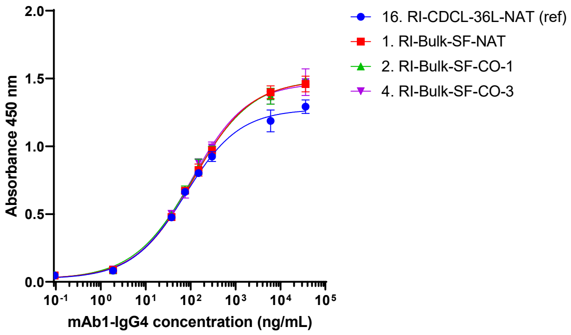
Absorbance 450 nm
mAb1-IgG4 concentration (ng/mL)
16. RI-CDCL-36L-NAT (ref)
1. RI-Bulk-SF-NAT
2. RI-Bulk-SF-CO-1
4. RI-Bulk-SF-CO-3

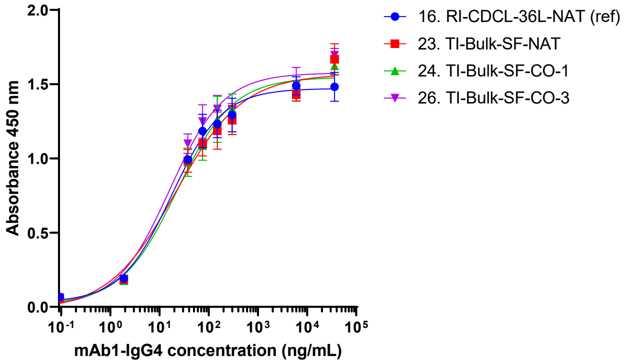
Absorbance 450 nm
mAb1-IgG4 concentration (ng/mL)
16. RI-CDCL-36L-NAT (ref)
23. TI-Bulk-SF-NAT
24. TI-Bulk-SF-CO-1
26. TI-Bulk-SF-CO-3

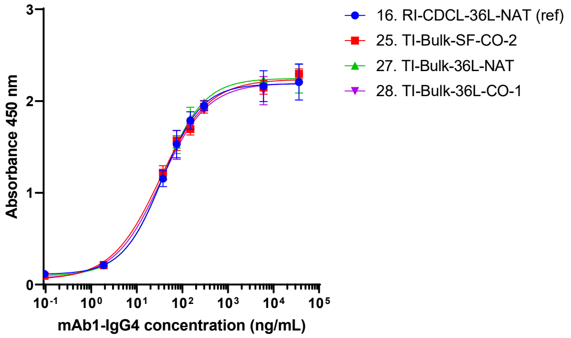
Absorbance 450 nm
mAb1-IgG4 concentration (ng/mL)
16. RI-CDCL-36L-NAT (ref)
25. TI-Bulk-SF-CO-2
27. TI-Bulk-36L-NAT
28. TI-Bulk-36L-CO-1

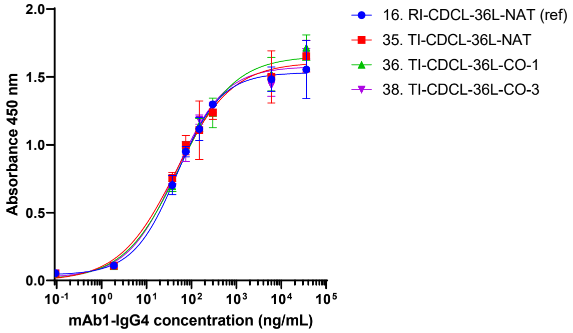
Absorbance 450 nm
mAb1-IgG4 concentration (ng/mL)
16. RI-CDCL-36L-NAT (ref)
35. TI-CDCL-36L-NAT
36. TI-CDCL-36L-CO-1
38. TI-CDCL-36L-CO-3

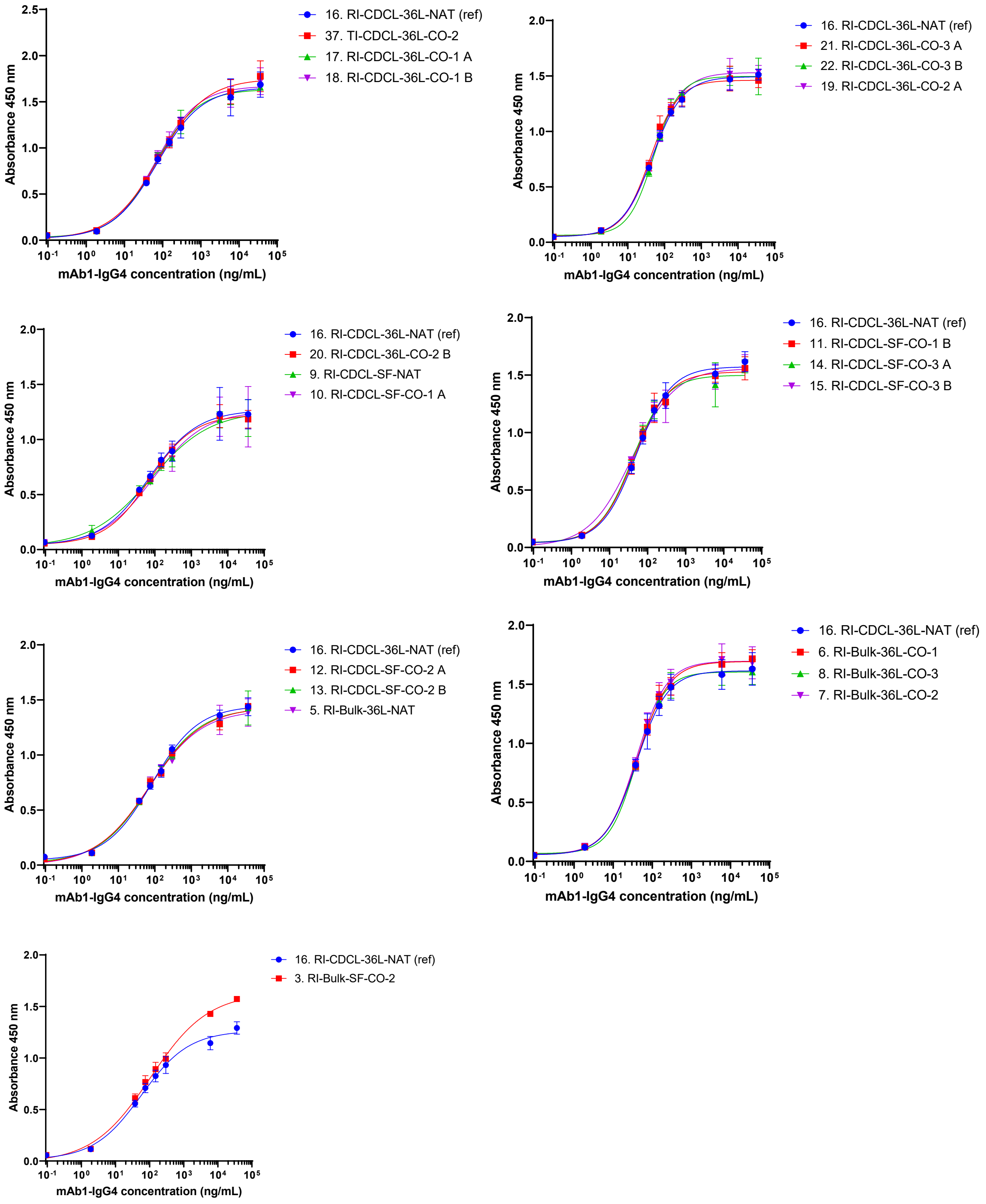

Supplementary Figure S6 ELISA dose response curves

Each ELISA setup allowed for triplicate measurement of one reference sample (#16) and three test samples. Each graph in this figure is representative of the three repeat ELISA plates measured for each set of three test samples. Compared to the reference sample, eight samples had higher upper asymptotes (#1-4 and #31-34). This observation is also reflected in the relative response at the highest dose reported in Fig. 6A.
