# Supplementary material for: Investigating the impact of synonymous gene recoding on a recombinantly expressed monoclonal antibody under different process parameters
Source: Bioeng Transl Med. 2025 Jan 27;10(3):e10750. doi: 10.1002/btm2.10750 (PMC12079346; doi:10.1002/btm2.10750)
Supplement: Supplementary file 4 — TABLE S1: CE‐SDS non‐reduced purity, fragments, aggregates. TABLE S2: CE‐SDS reduced purity, fragments aggregates, % non‐glycosylated HC. TABLE S3: SEC monomer, total fragments, total aggregates. TABLE S4: CE‐iCE main peak, acidic variants, basic variants. TABLE S5: Glycosylation—G0, G0F, G0F‐GlcNAc, G1F. TABLE S6: Glycosylation—G1F*, G1Fs, G2F, Man‐5. TABLE S7: Relative gene copy number (GCN), hydroxylamine species (HRS). TABLE S8: Titer, protein A capture, specific productivity. TABLE S9: KD, relative response at 36 μg/mL, relative potency. [file BTM2-10-e10750-s004.docx]

**Supplementary Document (Tables S1-S9):** Analysis of all product quality, binding kinetics, and ELISA response data

Question: Does the nucleotide (NT) sequence, cell line generation, cell line format, and production scale affect product quality of mAb1-IgG4 protein?

- 4 NT sequences (NTS): Native (NAT) and 3 differently codon optimized (CO-1, CO-2, CO-3)
- 2 cell line generation (CLG) methods: Random Integration (RI) or Targeted Integration (TI)
- 2 cell line formats (CLF): stable bulk culture (Bulk) or clonally derived cell lines (CDCLs)
- 2 production scales (PS): shake flask (SF) or 36L

Method: Manufacture mAb1-IgG4 protein via different combinations of these variables and perform product quality analysis (see manuscript for details about each analytical method). Perform statistical analysis of differences among samples by sorting into:

- Two groups based on [CLG], [CLF], or [PS]
- Four groups based on [NT sequence], [CLG and CLF], [CLG and PS], or [CLF and PS]
- Eight groups based on [CLG and NT], [CLF and NT], [PS and NT], or [CLG, CLF and PS].

Statistical analysis was performed among groups that did not have obvious subpopulations. If variances were visually consistent, a student’s t-test (two-tailed) was performed to analyze data comparing two groups (either CLG, CLF, or PS), an ordinary one-way analysis of variance (ANOVA) with Tukey’s multiple comparisons test was used to compare means in data comparing the four NT sequences, and a 2-way ANOVA with Tukey's multiple comparisons test was used to compare means among data from multiple variables. If variances were not visually consistent, Welch’s t-test (two-tailed) was performed. Significant differences between groups that differ in one variable are indicated on graphs: *p<0.05, **p<0.01, ***p<0.001, ****p<0.0001. Data are presented as mean +/- standard deviation (SD). All statistical analyses were performed using GraphPad Prism (version 9.5.1). Details for all results from statistical testing can be found in the Extended Statistics Excel file.

Relative GCN and specific productivity were not determined (n.d.) for some groups as indicated on relevant graphs. Select graphs are also included as figures in the manuscript.

Results:

[Table S1](#_Table_1): CE-SDS Non-Reduced Purity, Fragments, Aggregates

[Table S2](#_Table_2): CE-SDS Reduced Purity, Fragments Aggregates, % Non-glycosylated HC

[Table S3](#_Table_3): SEC Monomer, Total Fragments, Total Aggregates

[Table S4](#_Table_4): CE-iCE Main Peak, Acidic Variants, Basic Variants

[Table S5](#_Table_5): Glycosylation - G0, G0F, G0F-GlcNAc, G1F

[Table S6](#_Table_6): Glycosylation - G1F*, G1Fs, G2F, Man-5

[Table S7](#_Table_7): Relative Gene Copy Number (Rel GCN), Hydroxylamine Species (HRS)

[Table S8](#_Table_S8): Titer, Protein A Capture, Specific Productivity

[Table S9](#_Table_S9): KD, Relative Response at 36 µg/mL, Relative Potency

The results in these tables in conjunction with the translation kinetics and gel electrophoresis results, were used to draw the conclusions presented in the manuscript.

| Table S1: CE-SDS Non-reduced | | | |
| --- | --- | --- | --- |
|  | **Purity** | **Fragments** | **Aggregates** |
| 1 variable | | | |
| CLF |   ‡ |   ‡ |   ‡ |
| CLG |   ‡ |   ‡ |  |
| PS |   ‡ |   ‡ |   ‡ |
| NTS |   ns |   ns |   ‡ |
| 2 variables | | | |
| CLF vs. NTS |   ns |   ns |   ns |
| CLG vs. NTS |   ns |   ns |   ns |
| PS vs. NTS |   ns |   ns |   ns |
| CLG vs. CLF |   ‡ |   ‡ |  |
| CLF vs. PS |   ‡ |   ‡ |   ‡ |
| CLG vs. PS |   ‡ |   ‡ |   ‡ |
| 3 variables | | | |
| CLG vs. CLF vs. PS |  |  |  |

**Table S1**

‡: statistics not performed

ns: not significant

See Extended Statistics Excel file for details on statistical tests performed.

| Table S2: CE-SDS Reduced | | | | |
| --- | --- | --- | --- | --- |
|  | **Purity** | **Fragments** | **Aggregates** | **% Non-glycosylated HC** |
| 1 variable | | | | |
| CLF |   ‡ |   ‡ |   ‡ |   ‡ |
| CLG |   ‡ |   ‡ |   ‡ |   ‡ |
| PS |   ‡ |   ‡ |   ‡ |   ‡ |
| NTS |   ns |   ns |   ns |   ns |
| 2 variables | | | | |
| CLF vs. NTS |   ‡ |   ‡ |   ‡ |   ‡ |
| CLG vs. NTS |   ‡ |   ‡ |   ‡ |   ‡ |
| PS vs. NTS |   ‡ |   ‡ |   ‡ |   ‡ |
| CLG vs. CLF |   ‡ |   ‡ |   ‡ |   ‡ |
| CLF vs. PS |   ‡ |   ‡ |   ‡ |   ‡ |
| CLG vs. PS |   ‡ |   ‡ |   ‡ |   ‡ |
| 3 variables | | | | |
| CLG vs. CLF vs. PS |  |  |  |  |

**Table S2**

‡: statistics not performed

ns: not significant

See Extended Statistics Excel file for details on statistical tests performed.

| Table S3: SEC | | | |
| --- | --- | --- | --- |
|  | **Monomer** | **Total Fragments** | **Total Aggregates** |
| 1 variable | | | |
| CLF |  |   ‡ |  |
| CLG |   ns |   ‡ |   ‡ |
| PS |   ‡ |   ‡ |  |
| NTS |   ns |   ns |   ns |
| 2 variables | | | |
| CLF vs. NTS |  |   ns |   ns |
| CLG vs. NTS |   ns |   ‡ |   ns |
| PS vs. NTS |   ns |   ‡ |   ns |
| CLG vs. CLF |  |   ‡ |  |
| CLF vs. PS |  | ‡ |  |
| CLG vs. PS | ‡ | ‡ |  |
| 3 variables | | | |
| CLG vs. CLF vs. PS |  |  |  |

**Table S3**

‡: statistics not performed

ns: not significant

See Extended Statistics Excel file for details on statistical tests performed.

| Table S4: CE-iCE | | | |
| --- | --- | --- | --- |
| 1 variable | | | |
|  | **Main Peak** | **Acidic Variants** | **Basic Variants** |
| CLF | ns | ‡ | ‡ |
| CLG |  | ns | ns |
| PS |  |  | ns |
| NTS | ns | ns | ns |
| 2 variables | | | |
| CLF vs. NTS | ns | ns | ns |
| CLG vs. NTS | ns | ns | ns |
| PS vs. NTS | ns | ns | ns |
| CLG vs. CLF |  | ‡ | ns |
| CLF vs. PS |  | ‡ | ns |
| CLG vs. PS |  | ‡ | ns |
| 3 variables | | | |
| CLG vs. CLF vs. PS |  |  |  |

**Table S4**

‡: statistics not performed

ns: not significant

See Extended Statistics Excel file for details on statistical tests performed.

| Table S5: Glycosylation | | | | |
| --- | --- | --- | --- | --- |
|  | **G0** | **G0F** | **G0F-GlcNAc** | **G1F** |
| 1 variable | | | | |
| CLF | ns | ‡ | ‡ | ‡ |
| CLG | ns | ‡ | ‡ | ‡ |
| PS | ns |  | ns | ‡ |
| NTS | ‡ | ns | ns | ns |
| 2 variables | | | | |
| CLF vs. NTS |  |  | ‡ | ns |
| CLG vs. NTS |  | ns | ‡ | ns |
| PS vs. NTS |  | ns | ns | ns |
| CLG vs. CLF | ns | ‡ |  | ‡ |
| CLF vs. PS | ns |  | ‡ | ‡ |
| CLG vs. PS | ‡ | ‡ | ‡ | ‡ |
| 3 variables | | | | |
| CLG vs. CLF vs. PS | ns |  |  |  |

**Table S5**

‡: statistics not performed

ns: not significant

See Extended Statistics Excel file for details on statistical tests performed.

| Table S6: Glycosylation | | | | |
| --- | --- | --- | --- | --- |
|  | **G1F*** | **G1Fs** | **G2F** | **Man-5** |
| 1 variable | | | | |
| CLF | ‡ | ‡ | ‡ |  |
| CLG |  | ‡ | ‡ | ‡ |
| PS | ‡ | ‡ | ‡ |  |
| NTS | ‡ | ‡ | ‡ | ‡ |
| 2 variables | | | | |
| CLF vs. NTS | ns | ns |  | ns |
| CLG vs. NTS | ns | ns | ns |  |
| PS vs. NTS | ns |  |  | ns |
| CLG vs. CLF | ‡ | ‡ | ‡ | ‡ |
| CLF vs. PS | ‡ |  |  |  |
| CLG vs. PS | ‡ | ‡ | ‡ |  |
| 3 variables | | | | |
| CLG vs. CLF vs. PS |  |  |  |  |

**Table S6**

‡: statistics not performed

ns: not significant

See Extended Statistics Excel file for details on statistical tests performed.

| Table S7: Relative Gene Copy Number (Rel. GCN) and Hydroxylamine Species (HRS) | | | |
| --- | --- | --- | --- |
|  | **Rel. GCN - HC** | **Rel. GCN - LC** | **HRS** |
| 1 variable | | | |
| CLF |  |  |  |
| CLG | ‡ | ‡ | ns |
| PS | ‡ | ‡ |  |
| NTS | ‡ | ‡ | ns |
| 2 variables | | | |
| CLF vs. NTS |  |  | ns |
| CLG vs. NTS | ‡ | ‡ | ns |
| PS vs. NTS | ‡ | ‡ |  |
| CLG vs. CLF |  |  | ns |
| CLF vs. PS |  |  |  |
| CLG vs. PS | ‡ | ‡ |  |
| 3 variables | | | |
| CLG vs. CLF vs. PS |  |  |  |

**Table S7**

‡: statistics not performed

nd: not determined

ns: not significant

See Extended Statistics Excel file for details on statistical tests performed.

| Table S8 | | | |
| --- | --- | --- | --- |
|  | **Titer** | **Protein A Capture** | **Specific Productivity** |
| 1 variable | | | |
| CLF |  | ns | ns |
| CLG |  | ns |  |
| PS | ns |  |  |
| NTS | ‡ | ns | ns |
| 2 variables | | | |
| CLF vs. NTS |  | ns | ‡ |
| CLG vs. NTS |  | ns | ‡ |
| PS vs. NTS | ns | ns |  |
| CLG vs. CLF |  |  |  |
| CLF vs. PS |  |  |  |
| CLG vs. PS |  |  |  |
| 3 variables | | | |
| CLG vs. CLF vs. PS |  |  |  |

**Table S8**

‡: statistics not performed

nd: not determined

ns: not significant

See Extended Statistics Excel file for details on statistical tests performed.

| Table S9 | | | |
| --- | --- | --- | --- |
|  | Binding | ELISA relative to reference sample (RI-CDCL-36L-NAT) | |
|  | KD | Relative response at 36 ug/mL | Relative Potency |
| 1 variable | | | |
| CLF | ns |  | ns |
| CLG |  |  | ns |
| PS | ns |  | ns |
| NTS | ns | ns | ns |
| 2 variables | | | |
| CLF vs. NTS | ns |  | ns |
| CLG vs. NTS | ns |  | ns |
| PS vs. NTS | ns |  | ns |
| CLG vs. CLF | ns |  |  |
| CLF vs. PS | ns |  | ns |
| CLG vs. PS | ns |  | ns |
| 3 variables | | | |
| CLG vs. CLF vs. PS | ns |  |  |

**Table S9**

‡: statistics not performed

ns: not significant

See Extended Statistics Excel file for details on statistical tests performed.
