# Supplementary material for: Investigating the impact of synonymous gene recoding on a recombinantly expressed monoclonal antibody under different process parameters
Source: Bioeng Transl Med. 2025 Jan 27;10(3):e10750. doi: 10.1002/btm2.10750 (PMC12079346; doi:10.1002/btm2.10750)
Supplement: Supplementary file 5 — TABLE S10. Primer‐probe sequences of the qPCR assays. [file BTM2-10-e10750-s002.docx]

|  | **Forward primer (5'🡒3')** | **Reverse primer (5'🡒3')** | **Probe (5'🡒3')** | **Reporter Dye** |
| --- | --- | --- | --- | --- |
| CO-1 & CO-2  Light Chain | TGTGCCTGCTGAACAACTTCTAC | TGCTCGGTCACGGATTCC | AAGGTGGACAACGCCCTGCAGTCC | VIC |
| CO-1 & CO-2  Heavy Chain | AGTCCCGGTGGCAGGAA | GGACTTCTGGGTGTAGTGGTTGT | AACGTGTTCTCCTGCTCCGTGATGC | VIC |
| CO-3  Light Chain | CCAGGGAGGCCAAAGTACAG | TGACGCTCTCCTGGGAGTTC | AAGGTGGACAACGCCCTCCAATCG | VIC |
| CO-3  Heavy Chain | CGACGGCTCCTTCTTCCTCTA | GGACTTCTGGGTGTAGTGGTTGT | AACGTCTTCAGCTGCTCCGTGATGC | VIC |
| NAT-IgKC2 | CCTGACGCTGAGCAAAGCA | CAGGCCCTGATGGGTGACT | ACTACGAGAAACACAAAGTCTACGCCTGCG | VIC |
| NAT-IgG*CH3 | CTGACCTGCCTGGTCAAAGG | TTCTCCGGCTGCCCATT | CGACATCGCCGTGGAGTGGGA | VIC |
| CHO49 | TGGAGAGATGGCTCGAGGTT | TGGTTGCTGGGAATTGAACTC | AGAGCACCAACTGCTCTTCCAGAGGTCC | FAM |

**Supplementary Table S10. Primer-probe Sequences of the qPCR Assays**
